# Recent Progress of Two-Dimensional Thermoelectric Materials

**DOI:** 10.1007/s40820-020-0374-x

**Published:** 2020-01-23

**Authors:** Delong Li, Youning Gong, Yuexing Chen, Jiamei Lin, Qasim Khan, Yupeng Zhang, Yu Li, Han Zhang, Heping Xie

**Affiliations:** 1 Collaborative Innovation Centre for Optoelectronic Science & Technology, and Key Laboratory of Optoelectronic Devices and Systems of Ministry of Education and Guangdong Province, Institute of Microscale Optoelectronics, College of Chemistry and Environmental Engineering, Shenzhen University, Shenzhen, 518060 Guangdong People’s Republic of China; 2 Shenzhen Key Laboratory of Advanced Thin Films and Applications, College of Physics and Optoelectronic Engineering, Shenzhen University, Shenzhen, 518060 Guangdong People’s Republic of China; 3 Department of Mechanical and Mechatronics Engineering, University of Waterloo, Waterloo, ON Canada; 4 Shenzhen Key Laboratory of Special Functional Materials, Shenzhen Engineering Laboratory for Advanced Technology of Ceramics, Guangdong Research Center for Interfacial Engineering of Functional Materials, College of Materials Science and Engineering, Shenzhen University, Shenzhen, 518060 Guangdong People’s Republic of China; 5 Shenzhen Clean Energy Research Institute, Shenzhen University, Shenzhen, 518060 Guangdong People’s Republic of China

**Keywords:** Two-dimensional thermoelectric materials, Black phosphorus analogue, Tin selenide, Transition metal dichalcogenides, Photothermoelectric effect

## Abstract

Thermoelectric generators have attracted a wide research interest owing to their ability to directly convert heat into electrical power. Moreover, the thermoelectric properties of traditional inorganic and organic materials have been significantly improved over the past few decades. Among these compounds, layered two-dimensional (2D) materials, such as graphene, black phosphorus, transition metal dichalcogenides, IVA–VIA compounds, and MXenes, have generated a large research attention as a group of potentially high-performance thermoelectric materials. Due to their unique electronic, mechanical, thermal, and optoelectronic properties, thermoelectric devices based on such materials can be applied in a variety of applications. Herein, a comprehensive review on the development of 2D materials for thermoelectric applications, as well as theoretical simulations and experimental preparation, is presented. In addition, nanodevice and new applications of 2D thermoelectric materials are also introduced. At last, current challenges are discussed and several prospects in this field are proposed.

## Highlights

- A comprehensive review on the recent development of two-dimensional (2D) nanomaterials for bulk or thin-film thermoelectric materials, as well as composite filler, has been extensively presented.
- Development of micro-device platform and its application to study the inherent thermoelectric properties of individual single- and few-layer 2D nanomaterials.

## Graphical abstract

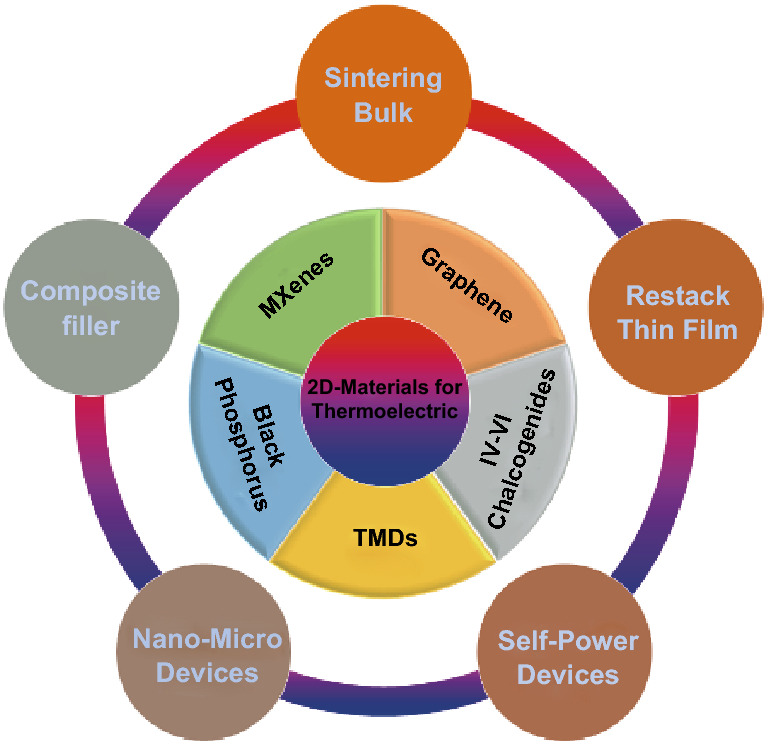

## Introduction

Due to the increasing demand in high-efficiency clean energy, it is essential to develop renewable energy devices to resolve the energy issues and avoid further environmental deterioration [1, 2]. In the past several decades, thermoelectric device, solar cells, wind-driven generators and fuel cells have attracted a large attention and shown a reliable quality in power generation. Among these kinds of energy conversion technologies, thermoelectric devices show a high potential in their application in many areas including power generators, cooling devices, and sensors [3–6]. Thermoelectric materials can directly convert thermal energy (such as waste heat and solar energy) into electrical energy; this property makes them important compounds for the development of sustainable energy efficient technologies [7–9]. When compared with other energy conversion devices, thermoelectric device show unique advantages including stability, long service life, and noiseless [10–12]. The thermoelectric performance of the materials at a certain temperature is evaluated by the dimensionless figure of merit (ZT), ZT=* σS*^2^*T*/*κ*. In this expression, *σ*, *T*, and *κ* correspond to the electrical conductivity, the Seebeck coefficient, and the absolute temperature, respectively. Moreover, *κ*_L_ refers to the lattice thermal conductivity *κ*_(L)_ and *κ*_(e)_ to the electronic thermal conductivity [13, 14]. These parameters are strongly coupled and dependent on the material’s band and crystal structure. Due to the complex inter-relation among these three parameters, it is almost impossible to optimize them independently [15]. However, several records for the highest ZT values have been continuously broken in the last few years due to the development of novel materials, new processing techniques, and new concept/mechanisms [16–20].

Since the initial discovery of graphene in 2004, the research interest in these materials has been growing explosively in the last decades [21, 22]. With the development of novel theoretical simulation methods and materials synthesis technics, a variety of 2D materials have been theoretically predicted and successfully fabricated. Typical 2D materials are graphene, black phosphorus (BP), transition metal dichalcogenides (TMDCs) (e.g., MoS_2_, WS_2_, MoSe_2_, and MoTe_2_), Group IVA–VA compounds (e.g., SnSe, GeSe, and SnS), nitrides (e.g., boron nitride), MXenes (e.g., Ti_3_C_2_, and Ti_4_N_3_), and Xenes (e.g., black phosphorene, arsenene, bismuthine, and antimonene) [21, 23–34] These 2D materials exhibit different allotropes with outstanding electronic and optical properties. For this reason, they have been widely used in electronics, optoelectronics, topological spintronic, bio-application energy storage (e.g., battery and supercapacitors) and energy conversion devices (e.g., thermoelectric and solar cells) [35–38].

Due to their outstanding advantages in electronic and mechanical properties, 2D materials with a layered structure have attracted a considerable attention as efficient thermoelectric materials [8, 39]. In the past several decades, the thermoelectric performance of a series of 2D materials, such as SnSe, Bi_2_Te_3_, and MoS_2_, has been theoretically predicted and the samples have been experimentally fabricated [33, 34, 40]. These 2D materials exhibit fascinating properties exhibit such as a large potential when they are used in the fabrication of high-performance thermoelectric devices.

Currently, although interesting research results in both theoretical predictions and experimental analyses have been achieved, a comprehensive review about 2D thermoelectric materials is still missing. To promote their development, it is pivotal to focus on the research progress in this field. In this review, the theoretical and experimental advances in the 2D thermoelectric materials field are summarized. Initially, their unique electrical and thermal properties are illustrated. Then, the application of 2D nanomaterials to fabricate bulk thermoelectric compounds, thin-film thermoelectric materials, and composite fillers is discussed in detail. Moreover, the thermoelectric properties of single- or multilayer 2D materials studied by using nano–micro-devices are introduced. Finally, thermoelectric compounds combined with photodetection devices are discussed. A perspective and an outlook on 2D thermoelectric materials conclude this review.

## Properties of 2D Thermoelectric Materials

### Graphene

As a typical 2D material, graphene has become a popular topic in scientific research due to its distinctive physical and chemical properties since it can be exfoliated from bulk graphite [41, 42]. Due to its unique electrical, optical, catalytic, and mechanical features, graphene has attracted a broad attention in recent years in many fields. For instance, graphene shows an ultrahigh electrical conductivity (10^6^ S cm^−1^) at room temperature due to its high electron mobility [43]. Its maximum Seebeck coefficient value was reported to be about 80 mV K^−1^ [27, 44, 45]. The thermal conductivity of graphene is in the 4840–5300 W mK^−1^ range at room temperature [46]. A large theoretical and experimental effort has been done to study the thermoelectric performance of graphene [44, 45, 47–69].

**Fig. 1:**
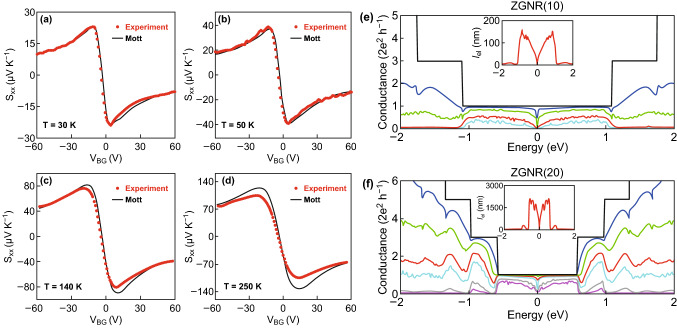
The in-plane Seebeck coefficient of bilayer graphene as a function of the backgate voltage (*V*_BG_) at different temperatures: **a** *T* = 30 K, **b** *T* = 50 K, **c** *T* = 140 K and, **d** *T* = 250 K. Reproduced with permission from Ref. [49]. Copyright 2010, American Physical Society. Electron transport through the edge-disordered zigzag edges graphene nanoribbons: sample lengths **e** Nz = 10 and **f** Nz = 20. Reproduced with permission from Ref. [50]. Copyright 2010, American Physical Society

Nam et al. investigated the in-plane thermoelectric properties of bilayer graphene by using a micro-device, and their results were simulated by using the Mott formula with a hyperbolic dispersion relation [49]. The gate-voltage dependence of the thermoelectric properties of bilayer graphene has been carried out for various temperatures and charge-carrier densities. Figure 1a–d shows the measured Seebeck coefficient of bilayer graphene, which follows the semiclassical Mott formula at low temperature (*T* = 30 K and *T* = 50 K). However, at high temperature (*T* = 140 K and *T* = 250 K), the Seebeck coefficient measured by using the micro-device reveals a deviation from the simulated Seebeck coefficient near the charge neutrality point, and this deviation increases with the temperature [49]. Figure 1e, f shows the simulation of the electron and phonon transport through an edge-disordered zigzag graphene layer [50]. The simulation results on the graphene nanoribbons with different sample lengths and number of zigzag chains (*N*z) were studied. Sevincli et al. found that the edge disorder in zigzag graphene nanoribbons (ZGNRs) can significantly reduce the phonon thermal conductance, whereas the electronic conduction remains almost intact at the first conductance plateau [50]. Sevincli predicted that the edge disorder suppresses the thermal conductivity by few orders of magnitude in the zigzag edges graphene nanoribbons. Moreover, it gives rise to a ZT value of 4 at 300 K [50].

These results indicate that graphene can be optimized to achieve high-performance thermoelectric properties. However, for practical applications, both the Seebeck coefficient and the power factor of graphene should be improved, whereas its thermal conductivity should be decreased. To achieve large ZT values in graphene-based thermoelectric materials, two major barriers need to be overcome: (1) the graphene thermal conductivity is too high, and (2) the Seebeck coefficient is too small due to its gapless band structure. Although graphene exhibits advantage as a high-performance thermoelectric material, more efforts in simulations and experiments need to be done to optimize its thermoelectric performance.

### TMDC

As a novel class of layered materials, 2D TMDCs, such as MX_2_ (M = Mo, W, Ti, and X = S, Se, Te), have attracted a large attention in last decades due to their semiconducting characteristics, outstanding chemical stability, and mechanical and physical properties. Moreover, they have been widely studied in various fields including optoelectronic, energy harvests and conversion, and cancer therapy. These types of material have been widely studied in many fields, such as photodetector, thermoelectric, and gas-sensing applications. Their relatively high electrical conductivity and relatively low thermal conductivity make the TMDCs emerge as promising materials for high-performance thermoelectric devices, especially for the fabrication of wearable heating/cooling devices and power generators.

The thermoelectric properties of 2D TMDCs with different thickness have been investigated both experimentally and theoretically. The most common TMDCs, such as MoS_2_, MoSe_2_, WS_2_, and WSe_2_, exhibit very similar electronic properties. For instance, the MoS_2_ monolayer is a direct semiconductor with a band gap of 1.9 eV, whereas bulk MoS_2_ is an indirect semiconductor with a band gap of 1.2 eV [70]. The band structures of the 2D TMDCs share a similar transition from the direct band gap to the indirect band gap, as the atomic layer increases from a monolayer to a bilayer [8].

**Fig. 2:**
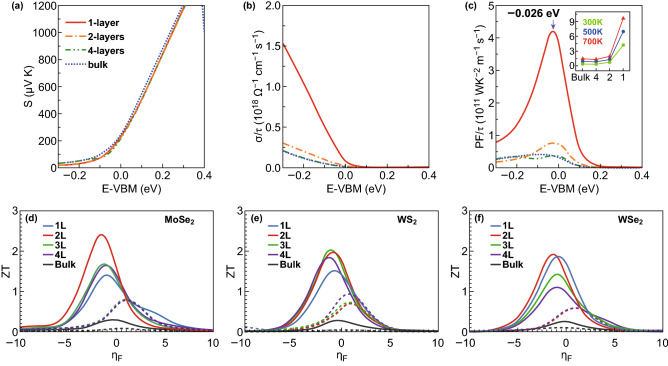
**a** In-plane Seebeck coefficient, **b** electrical conductivity, and **c** power factor of p-type MoS_2_ at 300 K depending on their thickness and chemical potential. Reproduced with permission from Ref. [73]. Copyright 2016, American Physical Society. ZT at 300 K for **d** MoSe_2_, **e** WS_2_, and **f** WSe_2_ for bulk (black), 1 layer (blue), 2 layers (red), 3 layers (green), and 4 layers (purple) structures as a function of the reduced Fermi energy, *η*_F_. The *n*-type ZT is plotted with a solid line and *p*-type ZT with a broken line. Reproduced with permission from Ref. [90] Copyright 2014, AIP Publishing. (Color figure online)

As previously mentioned, the ZT value depends on three inherent physical quantities: the thermal conductivity, the electrical conductivity, and the Seebeck coefficient. These quantities have been widely studied in the case of 2D materials. Owing to their characteristic quasi-two- dimensional crystal structures, TMDCs exhibit high Seebeck coefficient [71–73]. By considering MoS_2_ as a reference compound, a brief introduction about the thermoelectric properties of 2D TMDCs is presented below. As shown in Fig. 2a–c, the thermoelectric properties of MoS_2_ multilayers at 300 K are calculated along the in-plane direction. According to the calculation results, the Seebeck coefficients are not affected so much by the change of the layer numbers. However, the electric conductivity shows clear changes depending on the layer thickness, as shown in Fig. 2b. The significantly increased electrical conductivity as well as the *PF*/*τ* is mainly caused by the valley degeneracy at the valence band edge [73]. The ZT values of TMDCs (MoSe_2_, WS_2_, and WSe_2_) along the in-plane direction have been predicted. As shown in Fig. 2d–f, the few layers’ structure shows large increase in the values of their ZT compared to those of the bulk. Besides the theory prediction, the thermoelectric properties of 2D TMDCs have also been measured and the experimental results have proven that their thermoelectric properties can be enhanced by applying external electric field and pressure. Buscema et al. [71] reported a large Seebeck coefficient with a large tunability between − 4 × 10^2^ and − 1 × 10^5^ μV K^−1^ for the MoS_2_ monolayer in the presence of an external electric field. However, in a highly positive gate range, the Seebeck coefficient varies from −2 × 10^2^ to − 1.5 × 10^3^ μV K^−1^ and in a highly negative gate range, it varies from − 3 × 10^4^ to − 3 × 10^5^ μV K^−1^, showing an increases of about two orders of magnitude. Wu et al. observed a large Seebeck coefficient of 30 mV K^−1^ by adjusting the backgate voltage [74]. The thermoelectric properties of an exfoliated 2D MoS_2_ flake with different thickness were studied by Hippalgaonkar. Due to the high electron concentration of *n* = 1.06 × 10^13^ cm^−2^, the bilayer MoS_2_ exhibits the highest power factor (8.5 mW mK^−2^) as the gate voltage is 104 V [72]. Due to the high electron concentration of *n* = 1.06 × 10^13^ cm^−2^, the MoS_2_ bilayer exhibits the highest power factor (8.5 mW mK^−2^) as the gate voltage reaches 104 V.

The 2D TMDCs exhibit a relatively lower thermal conductivity when compared to graphene, which make them a promising material for various applications in thermoelectric devices. The low thermal conductivity of the 2D TMDCs has been widely studied in experimental and theoretical works [75–89]. For instance, Sahoo et al. measured the thermal conductivity of a suspended few-layer MoS_2_ film by using a laser-power-dependent Raman scattering experiment method. As a result, the thermal conductivity at room temperature for few-layer MoS_2_ was found to be 52 W mK^−1^ [80]. Yan et al. [79] carefully measured and analyzed the temperature-dependent Raman spectra of the exfoliated MoS_2_ monolayer at room temperature, and the measured thermal conductivity is 34.5 W mK^−1^. Taube et al. measured the temperature-dependent thermal conductivity of the MoS_2_ monolayer on a SiO_2_/Si substrate via Raman spectroscopy. The results show that the thermal conductivity decreases from 62.2 to 7.45 W mK^−1^ as the temperature increases from 300 to 450 K [78].

In addition, the wide tunability of inherent thermoelectric parameters of a MoS_2_ monolayer or a few layers makes this material an ideal candidate for applications as a conventional thermoelectric generator or a cooler. Although the calculation predictions show that the TMDCs should exhibit an outstanding thermoelectric performance, these results have not been proven yet. The measured Seebeck coefficient is much lower than the calculations suggest data, and the thermal conductivity is much higher than in the case of conventional thermoelectric materials but much lower than graphene.

### Group IVA–VIA Compounds

As the most efficient kinds of thermoelectric materials, the compounds that belong to group IVA–VIA have became the hot research field of thermoelectric science during the past decades. These materials, such as Sn(S, Se, T(e), Ge(S, Se, Te), Pb(S, Se, Te), Sn(Se, S)_2_, and their alloys, have been widely studied in the thermoelectric field. Moreover, they have been used to fabricate high-performance thermoelectric power generators, thermoelectric sensors, and cooling devices [91, 92]. Among them, Sn(S, Se) and Ge(S, Se), which belong to the space group Pnma, exhibit a typical layered 2D structure feature along the *c* direction. The reduction in the dimensionality of these materials has been proved as one of the most efficient methods to enhance their ZT values since the Seebeck coefficient increases due to the increased density of the states near the Fermi level [93, 94]. In 1993, by carefully studying the properties and the structure of a low-dimensional Bi_2_Te_3_ material, a theory to explain the relation between its structure and its thermoelectric properties was presented by Dresselhaus and Hicks [95]. According to their results, the numerous grain boundary and interfaces in the material would generate a strong phonon scattering, which leads to a reduction in its thermal conductivity. Because of this reduced thermal conductivity and almost unchanged electrical properties, an enhanced ZT value could be obtained in the case of such low-dimensional materials [95]. Several years later, Sofo and Mahan [96] proposed a modified theory. They suggested that the well-quantum mixing and the changing density of state from a 2D into a 3D lead to a higher thermoelectric performance. Subsequently, by converting them into the well-quantum, the highest ZT value of bulk Bi_2_Te_3_ was improved 13 times. This result confirms the validity of the theory that the reduction in the dimensionality is helpful to enhance the energy conversion efficiency of these materials [97]. Fei and Cheng [98, 99] reported a bismuth monolayer showing very promising thermoelectric properties.

As a typical compound belonging to the group IVA–VIA, tin selenide (SnSe) has been widely studied due to its outstanding electronic and photonic properties. SnSe exhibits potential applications in many fields such as in energy storage and conversion devices and novel optoelectronic devices [92, 100–102] SnSe exhibits a layered orthorhombic structure and belongs to the Pnma space group at room temperature. Its structure can be derived from a three-dimensional distortion of a rock-salt structure. Normally, the Sn and Se atoms are arranged in double layers with two planes consisting of zigzag Sn–Se chains along the a-axis.

As a thermoelectric material, the thermoelectric properties of SnSe are limited by its poor electrical conductivity at room temperature. However, Zhao et al. reported a remarkably high ZT value of 2.6 (at 923 K) and of 2.2 (at 773 K) in *p*-type and *n*-type single-crystal SnSe, respectively [33, 103]. These results triggered the researchers in performing further studies on the thermoelectric properties of SnSe and its alloys. According to Zhao’s study, such excellent thermoelectric performances originate from the ultralow thermal conductivity when the SnSe transfers into a high-temperature phase at *T *>973 K. Inspired by the ultralow thermal conductivity and by the excellent electrical properties, SnSe and its alloys have attracted a significant attention in recent years [104]. Despite the excellent thermoelectric properties obtained for the SnSe single crystals, their complex crystal growth conditions and high production costs limit their practical applications [105]. Therefore, scientists focused on the development of high-performance polycrystalline SnSe. Via systematic optimization methods, such as texturing, doping, and alloying, a significant enhancement of the ZT value of polycrystalline SnSe materials was achieved during the last several years [106–108]. Recently, the thermoelectric properties of polycrystalline SnSe have been improved continuously and their ZT value have been improved from 0.5 to nearly 1.7 via optimization of the carrier concentration [109, 110]. The thermoelectric performance of polycrystalline SnSe can further improved since its ZT value is still much lower than that of a single-crystal SnSe. For polycrystalline SnSe, the thermal conductivity remains higher than its theoretical calculation value and its electrical conductivity is lower than a single-crystal SnSe due to the existing grain boundaries [111]. Achieving an ultrahigh ZT value for the polycrystalline comparable to its single-crystal counterpart is still a challenge, and more experimental and theoretical work needs to be done in the next years.

### Black Phosphorus

A monolayer phosphorene and a few-layer phosphorene have been successfully exfoliated from bulk BP in 2014 and have attracted a tremendous research interest in the past few years [28, 112, 113]. As a novel elementary 2D material, the few-layer BP has gained tremendous attention in theoretical and experimental investigations [114–119]. Due to its unique crystal structure and in-plane anisotropic properties, BP has been widely studied in various fields including in the development of photodetectors [116, 120, 121], cancer therapies [115, 122, 123], supercapacitors [124], field-effect transistors (FETs) [125, 126], batteries [127, 128], and thermoelectric devices [39, 98, 129–133].

**Fig. 3:**
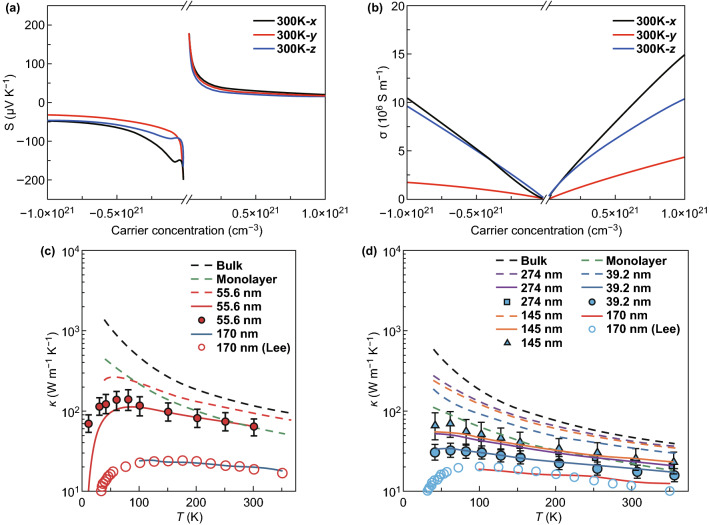
**a** Calculated Seebeck coefficient and **b** electrical conductivity of a few-layer black phosphorus at *T* = 300 K. Reproduced with permission from Ref. [132]. Copyright 2016, Royal Society of Chemistry. Measured thermal conductivity of black phosphorus depending on their thickness and temperature along different directions: **c** zigzag direction, **d** armchair direction. The dashed lines and the solid lines correspond to the calculation results for the defect-free black phosphorus and the black phosphorus with additional vacancy point defects, respectively. Reproduced with permission from Ref. [135]. Copyright 2017, Wiley–VCH

Moreover, BP has also been reported as a prospective materials for the production of thermoelectric devices due to its large Seebeck coefficient (335 μV K^−1^ at room temperature) [130], high carrier mobility (1000 cm^2^ (Vs)^−1^ at room temperature) [28], and moderate band gap (0.3–2.0 eV) [28, 112, 130]. According to the experimental results, bulk BP displays a high Seebeck coefficient of 335 μV K^−1^ and the few-layer BP show an increased Seebeck coefficient up to 510 μV K^−1^ [130, 134]. Due to its puckered structure, the electrical conductivity, the Seebeck coefficient, and the thermal conductivity of BP exhibit a strongly in-plane anisotropic behavior. This characteristic has been proved theoretically and experimentally in various kinds of BP materials including few-layer BP, thin-film BP, and bulk BP [135–140]. Recently, a number of theoretical simulation results have been published and predicted that the few-layer BP is an appealing materials with outstanding thermoelectric properties. As shown in Fig. 3a, b, the Seebeck coefficient and the electrical conductivity were calculated by using the first-principles calculations and the Boltzmann transport theory. These results are presented as a function of the carrier concentration at 300 K [132]. As predicted, a peak ZT value of 1.1 was obtained with an electron concentration of 1.5 × 10^20^ cm^−3^. The electrical conductivity of the few-layer BP along the zigzag direction is much lower than along the armchair direction. The Seebeck coefficient almost maintains an identical level along all the directions. The thermal conductivities of the few-layer BP with different thicknesses are shown in Fig. 3c, d. It can be concluded that the thermal conductivity along the zigzag direction is higher than that along the armchair direction. Moreover, the ZT value can be further enhanced by doping the few-layer BP with a proper element. Zhang et al. [132] investigated the thermoelectric properties of the Sb-doped few-layer BP, and their calculation indicates that the highest ZT value measures almost 6.0 for P_0.75_Sb_0.25_. As shown in Fig. 3, the anisotropy of the electrical conductivity corresponds to the opposite of its thermal conductivity, which makes the study of BP for thermoelectric more challenging [39]. Although the theoretical results have predicted that BP, the few-layer BP, and bulk BP exhibit a huge potential as excellent thermoelectric materials, only a few experimental results were reported. A larger experimental efforts need to be done to promote layered BP as a potential candidate for the production of thermoelectric devices.

### MXene

As a newly developed group of 2D materials consisting of transition metal carbides and nitrides, MXenes exhibit either metallic or semiconducting properties depending on its surface functionalization [141]. MXenes can be prepared by exfoliating the MAX phases into 2D nanosheets via selective etching of “A” layers by using appropriate hydrofluoric acids [29, 141–145]. These materials are defined as M_*n*+1_AX_*n*_, where *n* = 1, 2, or 3, “M” corresponds to an early transition metal (Sc, Ti, V, Cr, Zr, Nb, Mo, Hf, Ta), “A” is an element from groups 13–16 in the periodic table (Al, Si, P, S, Ga, Ge, As, In, Sn, Tl, Pb), and “*X*” is carbon and/or nitrogen. Due to its excellent thermal stability in air, MXene has been studied as a high-temperature thermoelectric material. Moreover, Kumar et al. have predicted that the functionalization of MXene by O, F, and OH groups can significantly affect its structural properties and electronic band structure, further enhancing its thermoelectric performance [146]. For instance, when ScC_2_ is functionalized with C, O, or OH, its band gap measures 1.84 (indirect), 1.03 (indirect), and 0.44 eV (indirect), respectively [146]. Such varied band gap structure leads to different thermoelectric properties of the materials. The calculation results show that the Seebeck coefficient of Sc_2_C(OH)_2_ measures only 372 μV K^−1^, while the Seebeck coefficient of Sc_2_CO_2_ and Sc_2_CF_2_ is above 1000 μV K^−1^ at room temperature [146]. Furthermore, the calculation results show that these materials exhibit different thermal conductivity. The lattice thermal conductivity of Sc_2_CO_2_, Sc_2_CF_2_, and Sc_2_C(OH)_2_ is 59, 36, and 10 W mK^−1^, respectively. By using the Boltzmann transport theory and the first-principles electronic structure calculations, Khazaei et al. [141] predicted the thermoelectric properties of more than 35 kinds of different functionalized MXene monolayers and multilayers of the type M_2_C, where M=Sc, Ti, V, Zr, Nb, Mo, Hf, and Ta, and M_2_N, M=Ti, Zr, and Hf. The calculation results prove that the monolayer and multilayer Mo_2_C nanosheets exhibit a higher power factor than other functionalized MXenes samples. However, several positive results show that MXene can achieve a higher thermoelectric performance. According to the calculations, almost all the semiconducting MXene shows a Seebeck coefficient larger than 100 μV K^−1^ at 400 K and some of them (such as Mo_2_CF_2_, Mo_2_C(OH)_2_, and Mo_2_CCl_2_) exhibit a high electrical conductivity [141].

The outstanding thermoelectric performance of MXenes has also been proved by a series of experiments. Kim et al. fabricated two kinds of Mo-based MXene (Mo_2_CT_*x*_, Mo_2_TiC_2_T_*x*_, and Mo_2_Ti_2_C_3_T_*x*_) flexible thin films and studied their thermoelectric properties [147]. For instance, the Mo_2_TiC_2_T_*x*_ film exhibits the highest power factor (about 309 μW mK^−2^ at 800 K) among these samples. Although a high thermoelectric performance for MXene has been theoretically predicted [141, 146–148], more experimental results have to be performed.

## Thermoelectric Materials Based on 2D Materials

Layered 2D materials have been widely employed as efficient thermoelectric materials in the last decades. In this section, the 2D layer structure materials based on the elements belonging to the groups IV–VI and on TMDCs are investigated. The previously reported results proved that bulk and monolayer materials exhibit extremely different thermoelectric properties [149]. In this section, the thermoelectric properties of bulk and thin films based on 2D materials are discussed.

### Bulk Thermoelectric Materials

Due to the limitations in materials synthesis techniques, bulk thermoelectric materials are usually fabricated via a simple melting process or a ball milling process followed by a post-sintering process (such as spark plasma sintering or hot press process). Although the materials exhibit a layer-by-layer structure, the synthesis of these compounds via these methods is not discussed in this review. With the development of nanotechniques, a series of new methods have been developed to fabricate nanomaterials with a variety of microstructures. Currently, it is possible to synthesize 2D compounds in large amounts to fabricate bulk thermoelectric materials. Since the grain boundary can scatter the phonons and can lead to low a thermal conductivity, it may be useful to fabricate high-performance thermoelectric materials by decreasing the particle size of the nanostructure thermoelectric material. Recently, 2D metal chalcogenides (including the group IV–VI compounds and the TMDCs) have triggered a considerable attention in the field of thermoelectrics as they exhibit a high ZT value. Due to their anisotropic crystal and electronic structures, these materials exhibit an intrinsically low thermal conductivity, which makes them promising thermoelectric materials [17, 150].

**Fig. 4:**
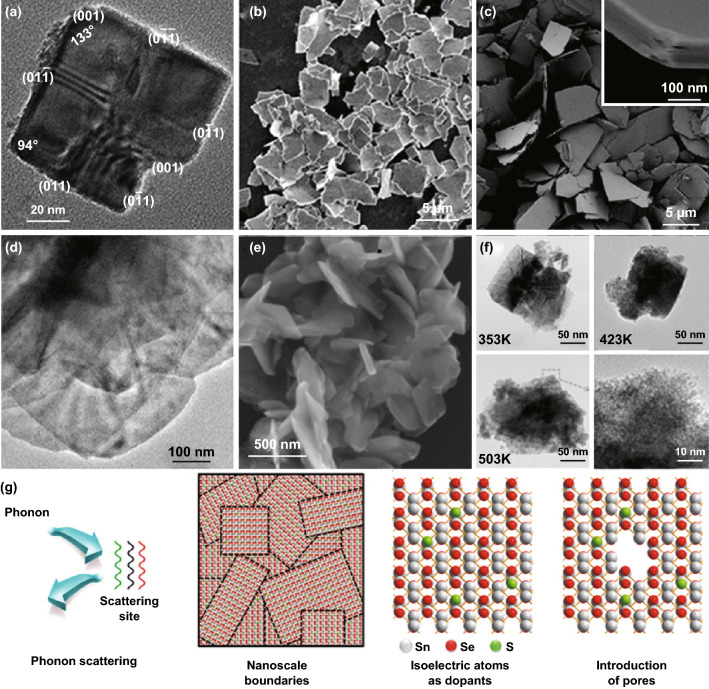
**a** TEM image of a SnSe nanoplate. Reproduced with permission from Ref. [151]. Copyright 2016, Wiley–VCH. **b** SEM image of the SnSe nanomaterials. Reproduced with permission from Ref. [152]. Copyright 2019, Wiley–VCH. **c** SEM image of the SnSe_0.9_Te_0.1_ nanoplates. The inset shows the layer thickness. Reproduced with permission from Ref. [154]. Copyright 2017, Royal Society of Chemistry. **d** TEM image of the SnSe nanosheets. **e** SEM image of the Sn_0.94_Bi_0.06_Se nanosheets. Reproduced with permission from Ref. [150]. Copyright 2018, American Chemical Society. **f** Low-magnification FE-TEM images of the porous SnSe_0.8_S_0.2_ NSs treated at the reaction temperatures of 353, 423, and 503 K and high-magnification FE-TEM image of the sample in the panel. **g** Schematic diagram of the phonon scattering mechanism of the SnSe_0.8_S_0.2_ NSs. Reproduced with permission from Ref. [153]. Copyright 2017, American Chemical Society

Among the 2D metal chalcogenides thermoelectric materials, SnSe seems to be promising commercially available thermoelectric material. Until now, the highest ZT value for both the *p*-type (2.6 at 923 K) and *n*-type (2.8 at 923 K) compounds was obtained from SnSe single crystals. This may be due to their layered structure, soft chemical bonding, and lattice anharmonicity. However, the ZT value of polycrystalline SnSe bulk materials is lower than those of single-crystal ones. During the last several years, numerous efforts have been done to fabricate high-performance SnSe polycrystalline bulk thermoelectric materials. 2D SnSe samples with different morphologies can be synthesized by using different methods. Figure 4a–c shows typical 2D SnSe nanosheet and nanoplate morphologies [150–152]. For instance, Han et al. developed a surfactant-free solution-based method for the synthesis of single-phase SnSe nanoplates. Their power factor measured perpendicularly to the hot-pressing direction reaches 0.4 mW mK^−2^ at 550 K [151]. Rongione et al. [152] fabricated a series of SnSe nanosheets and obtained an ultralow thermal conductivity of 0.09 W m^−1^ K^−1^. Such value mainly originates from the large thermal boundary resistance of the materials due to the strong phonon scattering near the interfaces between two SnSe nanosheets. In order to further improve the ZT value of SnSe bulk, Bi-, Te-, and S- doped SnSe nanosheets were synthesized by using a large variety of methods [150, 153–155]. The SEM morphology of the doped SnSe samples is shown in Fig. 4d–f. Chandra et al. synthesized a 2D ultrathin *n*-type Bi-doped SnSe nanosheet via a simple low-temperature solvothermal method [150]. The thickness of the SnSe nanosheet measures about 1–3 nm. Due to the presence of the nanoscale grain boundaries and the layered anisotropic structure, the heat-carrying phonons strongly scatter. This leads to an ultralow lattice thermal conductivity of 0.3 W mK^−1^ in the range of 300–720 K. Ju et al. fabricated a porous SnSe_1−*x*_S_*x*_ nanosheet, and the details of this structure are shown in Fig. 4g [153]. A reduction in the thermal conductivity and an improved ZT value were obtained. Such enhanced ZT value is mostly due to the substitution of the S atoms into SnSe, which induces the scattering of the phonons and several atomic disorders and nanosized boundaries. The mechanism of phonon scattering in porous SnSe_0.8_S_0.2_-based materials is illustrated by the simple schematic diagram in Fig. 4g. As a result, the high ZT value of the bulk based on porous SnSe_0.8_S_0.2_ nanosheets reaches 0.12 at 310 K. The substitution of the S atoms into SnSe and the fabrication of the SnSe_1−*x*_S_*x*_ NSs reduce the thermal conductivity by introducing phonon scattering in the atomic disorders and nanoscale boundaries, leading to a higher ZT.

Similarly to SnSe, SnSe_2_ is also a layered structure thermoelectric material and exhibits promising thermoelectric performance [156–159]. Saha et al. fabricated ultrathin Cl-doped SnSe_2_ nanosheets with the thickness of 3–5 nm via a simple low-temperature solvothermal process [159]. Similarly to SnSe, the hot-pressed SnSe_2_ pellet achieves an ultralow thermal conductivity of 0.67 W/mK due to its effective phonon scattering at the grain boundary, which is induced by its anisotropic layered structure.

**Fig. 5:**
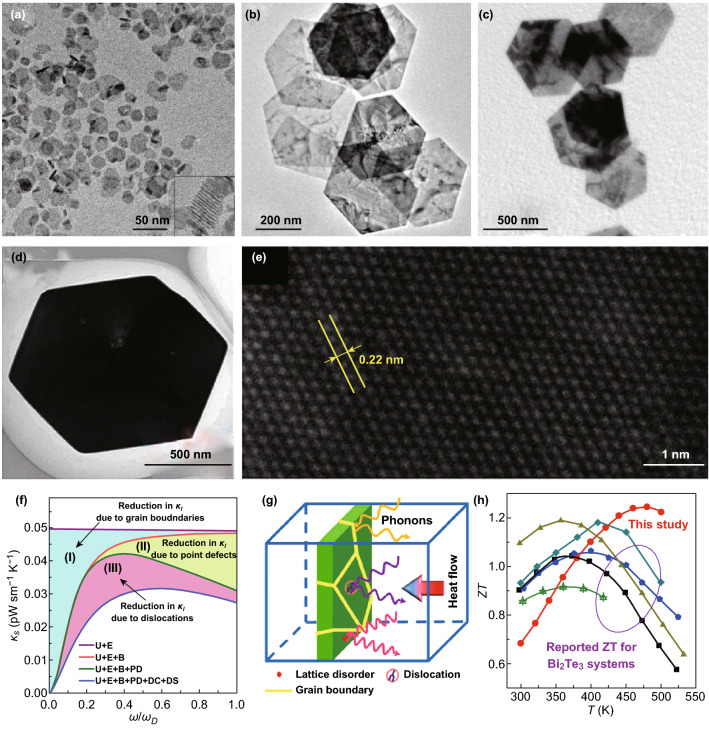
**a** TEM image of the Bi_2_Te_3_ nanoplate, the inset shows the lateral view of the stacked plates). Reproduced with permission from Ref. [161]. Copyright 2012, American Chemical Society. **b** Bright-field TEM image of the Bi_2_Se_3_@Bi_2_Te_3_@Bi_2_Se_3_ double shell nanoplates. **c** TEM image of the Bi_2_Se_3_@Bi_2_Te_3_@Bi_2_Se_3_@Bi_2_Te_3_ multishell nanoplates. Reproduced with permission from Ref. [162]. Copyright 2015, American Chemical Society. **d** TEM image, and **e** HRTEM image of the Bi_2_Te_2.7_Se_0.3_ nanoplate. **f** Calculated room-temperature *κ*_s_ value for the phonons scattering of the Bi_2_Te_2.7_Se_0.3_ pellet. **g** Schematic diagram illustrating the scattering of wide-frequency phonons by various sources. **h** Carriers mobility and ZT value of the Bi_2_Te_2.7_Se_0.3_ pellet. Reproduced with permission from Ref. [163]. Copyright 2016, American Chemical Society

During the last decades, the most efficient single-phase thermoelectric materials used in the near room temperature range are Bi_2_Te_3_-based alloys, which are also layered materials. Until now, the optimized ZT value of these compounds measure around 1.0. The highest ZT value for the p-type and n-type Bi_2_Te_3_ alloys is obtained in the case of Bi_0.5_Te_1.5_Se_3_ (ZT_max_ = 1.2) and Bi_2_Te_2.7_Se_0.3_ (ZT_max_ = 0.9) [17, 160]. Both these samples are bulk materials. Recently, Bi_2_Te_3_-based nanosheets have been widely reported in the fabrication of bulk Bi_2_Te_3_-based thermoelectric materials. As shown in Fig. 5a, Son et al. synthesized an ultrathin Bi_2_Te_3_ nanoplate with a thickness of about 1 nm via a simple solution process [161]. The Bi_2_Te_3_ bulk was prepared by sintering Bi_2_Te_3_ nanoplates via spark plasma sintering, and the maximum ZT value obtained was 0.62 at 400 K. Figure 5b, c shows the TEM images of the Bi_2_Se_3_@Bi_2_Te_3_ heterostructure nanoplates and of the Bi_2_Se_3_@Bi_2_Te_3_@Bi_2_Se_3_@Bi_2_Te_3_ multishell nanoplates, which were scalable synthesized via a solution epitaxial growth process [162]. The thickness of the nanoplate is in the 5–20 nm range. Via the simultaneous modulation of the electronic and thermal transport in the presence of highly dense grain and phase boundaries, the peak ZT value measures 0.71 and it was obtained at 450 K for via the bulk sintering of the Bi_2_Se_3_@Bi_2_Te_3_ nanoplates. Hong et al. synthesized a *n*-type Bi_2_Te_3−*x*_Se_*x*_ nanoplate by using the microwave-assisted surfactant-free solvothermal method. The TEM images of the nanoplate are shown in Fig. 5d, e [163]. The schematic diagram of the phonon scattering is shown in Fig. 5f, g. The grain boundaries, the point defects, and the dislocations may lead to the reduction in the lattice thermal conductivity. The experimental results prove these predictions. In this case, a relatively high ZT value of 1.23 at 480 K for the n-type Bi_2_Te_2.7_Se_0.3_ nanostructures was obtained from the sintered pellets. Since the nanostructure was employed as a raw material to obtain the sinter pellets, a high amount of grain boundaries was maintained and substantially reduced the thermal conductivity of the sample [163].

In the case of the bulk layered thermoelectric materials, the layered structure induces strong anisotropic thermoelectric properties along three different directions. More importantly, the grain boundaries, which were introduced by the nanostructuring, can substantially reduce the thermal conductivity when compared to the pellet structure. These samples were, in fact, synthesized by using ball milling and other melting methods. The 2D nanostructure materials employed to fabricate bulk thermoelectric materials further enhance their ZT value.

### Thin-Film Thermoelectric Materials

Thin-film thermoelectric materials have attracted a large attention due to their potential application as flexible and wearable devices. Besides the investigations on polymer-based thin-film thermoelectric materials, the more recent research efforts have been focused on thin-film thermoelectric materials fabricated by using nanostructures. Such newly developed 2D materials, such as graphene, BP, TMDCs, and group IV–VI compounds, can be easily synthesized and manufactured into thin films via various methods. As previously mentioned, the quantum size effect induced in the 2D nanostructure materials can increase the Seebeck coefficient. Moreover, the phonon scattering at the interfaces may lead to the a decrease in the thermal conductivity. The methods used to fabricate thin-film thermoelectric materials include vacuum evaporation, pulsed laser deposition, molecular beam epitaxy, magnetron sputtering, drop casting, spin coating, and inkjet printing [164–166].

**Fig. 6:**
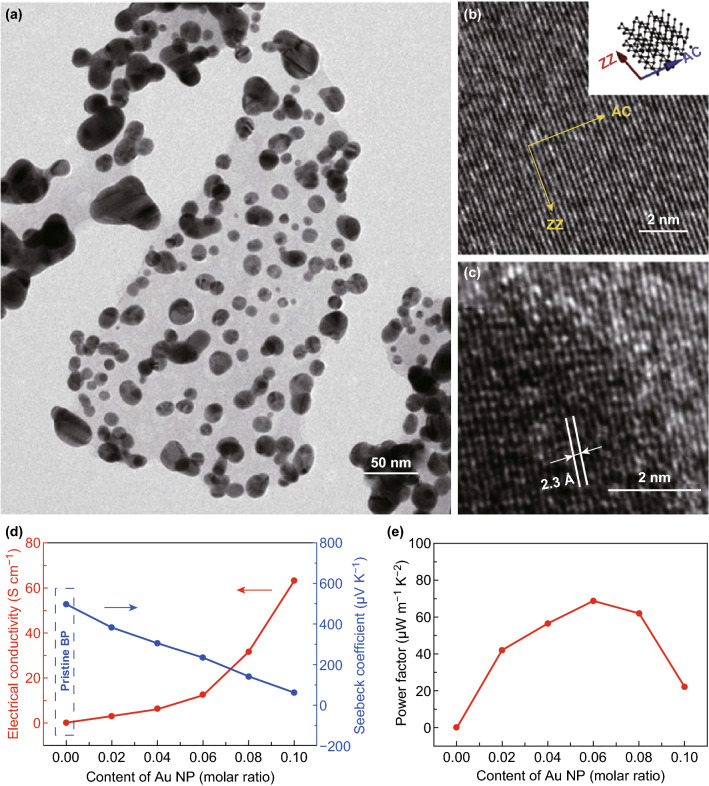
**a** Low-magnitude TEM image of the black phosphorus nanosheet decorated with Au nanoparticles. **b** Lattice image of the exfoliated black phosphorus nanosheet (inset illustration showing the corresponding crystal structure). **c** High magnitude HRTEM image of the black phosphorus nanosheet decorated with Au nanoparticles. **d** In-plane Seebeck coefficient, electrical conductivity, and **e** their corresponding in-plane power factor of the black phosphorus decorated with Au nanoparticles. Reproduced with permission from Ref. [129]. Copyright 2018, Wiley–VCH

Due to its unique electronic and optical properties, 2D BP has been widely studied in many research fields [114, 120, 122–124, 167–174]. Furthermore, it has been reported that it exhibits an ultrahigh Seebeck coefficient in the order of 10 mV K^−1^, which makes it a promising thermoelectric materials. Flores et al. studied the thermoelectric properties of bulk BP and found that its Seebeck coefficient measures about 335 μV K^−1^ at room temperature [130]. These results are lower than the theoretical predict data. Due to the poor stability of 2D BP, it is difficult to fabricate thermoelectric devices based on pristine BP. An et al. [129] decorated the BP surface with Au nanoparticles to enhance its thermoelectric properties and stability. The characterization of such structure is shown in Fig. 6a–c and its thermoelectric properties in Fig. 6d, e. The highest value of the Seebeck coefficient of this sample reaches 498 μV K^−1^ and the highest power factor is 68.5 μW mK^−2^, which is about 2740 times higher than that of pristine BP.

**Fig. 7:**
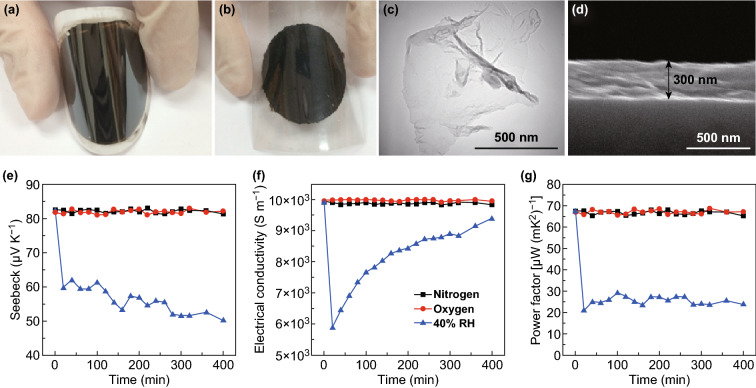
MoS_2_ nanosheets restacked film **a** on a cellulose ester membrane and **b** on a PET substrate. **c** TEM image of a single exfoliated MoS_2_ nanosheet and **d** cross-sectional SEM image of the MoS_2_ nanosheets restacked film. **e** Dynamic Seebeck coefficient, **f** electrical conductivity, and **g** power factor of the MoS_2_ nanosheets restacked film in a nitrogen, oxygen and 40% relative humidity atmosphere. Reproduced with permission from Ref. [181]. Copyright 2016, Elsevier

Recently, 2D TMDCs have collected a wide attention due to their special properties and are considered to be the next generation of high-performance thermoelectric materials [70, 74, 90, 175–180]. The measured Seebeck coefficient of single-layer MoS_2_ can measure over 30 mV K^−1^, providing an ideal candidate material for high-performance thermoelectric devices. Huang et al. studied the thermoelectric properties of metallic 1T-phase MoS_2_ nanosheets. These MoS_2_ nanosheets were synthesized via a chemically exfoliated process, and the thin films were fabricated via a simple vacuum-assisted filtration process, as shown in Fig. 7a–d [181]. The schematic diagram of the crystal structure and the band structure of the MoS_2_ monolayer were calculated. By studying the band structure of the 2H and of the 1T MoS_2_ monolayers, the high thermoelectric performance of the MoS_2_ film can be attributed to the metallic characteristic and to the conductivity nature of the 1T phase MoS_2_. As shown in Fig. 7e, g, the power factor of the MoS_2_ film reaches 73.1 μW mK^−2^ and exhibits an outstanding stability. However, the thermoelectric performance of MoS_2_ can be further enhanced by introducing several modifications. For instance, when the material is decorated with Au nanoparticles, the power factor of MoS_2_ increases to about 166.3 μW mK^−2^ [182].

**Fig. 8:**
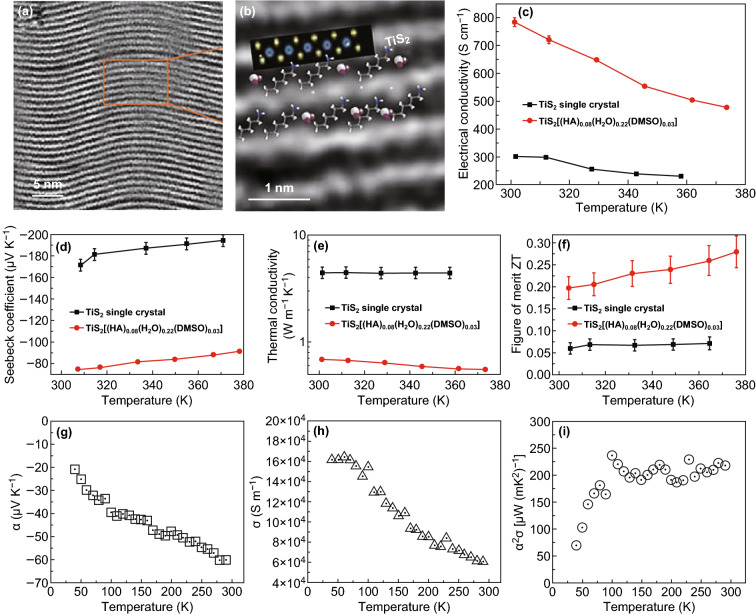
**a**, **b** HAADF-STEM image of the TiS_2_[(HA)_*x*_(H_2_O)_*y*_(DMSO)_*z*_] hybrid superlattice, **c** electrical conductivity, **d** Seebeck coefficient, **e** thermal conductivity, and **f** ZT of the TiS_2_[(HA)_*x*_(H_2_O)_*y*_(DMSO)_*z*_] hybrid superlattice. Reproduced with permission from Ref. [186]. Copyright 2015, Springer Nature. **g** Seebeck coefficient, **h** electrical conductivity, and **i** power factor of the TiS_2_ nanosheet assembled thin film. Reproduced with permission from Ref. [183]. Copyright 2017, American Chemical Society

TiS_2_ is another widely studied 2D thermoelectric materials [13, 183–186]. As shown in Fig. 8a, Wan et al. [186] introduced a novel electrochemical intercalation and solvent exchange process to synthesize a *n*-type TiS_2_[(hexylammonium)_*x*_(H_2_O)_*y*_(DMSO)_*z*_] hybrid superlattice. The organic cations were distributed onto the two sides of each TiS_2_ layer. As shown in Fig. 8c–f, an electrical conductivity of 790 S cm^−1^ and a very high power factor of 0.45 mW mK^−1^ were obtained in the case of the hybrid superlattice of TiS_2_/[(hexylammonium)_*x*_(H_2_O)_*y*_(DMSO)_*z*_]. Moreover, its in-plane lattice thermal conductivity measures about 0.12 W mK^−1^. This value is two orders of magnitude lower than that of single-layer and bulk TiS_2_. A high ZT value of 0.28 at 373 K was reported due to the high power factor and low thermal conductivity of the superlattice. This material exhibits an enormous potential for its application in wearable electronics devices. Tian et al. [184] fabricated a TiS_2_/organic superlattice and tried to manufacture a P–N prototype device. The devices with 5 P–N legs can generate an output voltage of 33 mV with a maximum power density of 2.5 W m^−2^. The thermoelectric performance of TiS_2_ can also be enhanced via a chemical welding process. As shown in Fig. 8g, i, when the TiS_2_ nanosheets bridge with the multivalent cationic metal, Al^3+^, during the film deposition process, the Seebeck coefficient and the electrical conductivity can be improved simultaneously, thus leading to an enhanced power factor [183].

## 2D Materials as Composite Filler

Composite engineering has been considered the simplest and most efficient way to enhance the performance of thermoelectric materials since the formative crystallite boundaries scatter phonons effectively and decrease the thermal conductivity of the material. Moreover, the transport properties can be optimized if the filler is distributed in the matrix with a proper amount. In addition, it is possible to decouple the three parameters and to enhance the thermoelectric performance of the materials.

### 2D Materials in Inorganic Bulk Thermoelectric Materials

The fabrication of hybrid composites is one of the most important methods to improve the thermoelectric performance of bulk thermoelectric materials, in which the nanomaterials are either located at the grain boundary or homogeneously dispersed in the matrix. The matrix materials form a continuous conductive network to maintain the same electrical conductivity within the matrix. Moreover, the dispersed second phase present in the matrix can scatter the phonons. The addition of a 2D material introduces a heterojunction interface, which can scatter the carrier and decrease its mobility, thus leading to a further enhancement of the thermoelectric performance. By adding a nanofiller added into the matrix, the relation among the electrical conductivity, the Seebeck coefficient, and the thermal conductivity can be decoupled by modulating the electron and phonon-transport characteristics.

In order to further enhance the thermoelectric properties of conventional thermoelectric materials, several kinds of 2D materials have been added into the bulk to fabricate different composites. Upon the addition of 2D materials filler, the carrier and the phonon-transport characteristics can be modulated. Besides, the addition of a 2D material introduces a heterojunction interface, which can scatter the carrier and decrease its carrier mobility, thus leading to an enhancement in the thermoelectric performance of the compound.

**Fig. 9:**
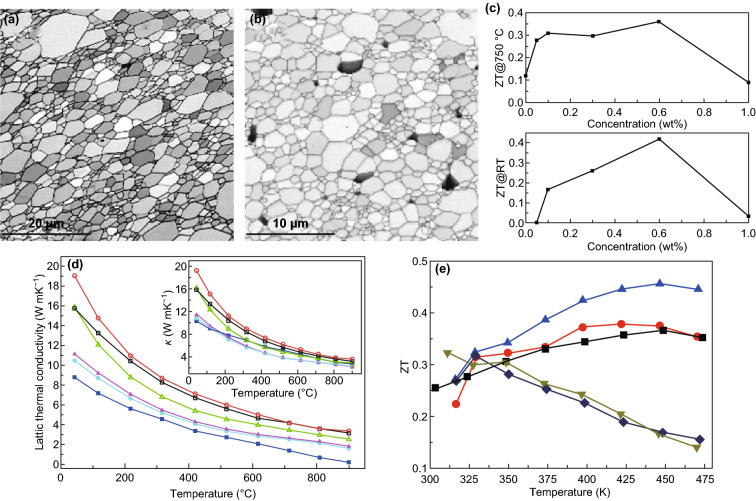
EBSD analysis of lanthanum strontium titanium oxide sample, and graphene/lanthanum strontium titanium oxide. **a** Band contrast image of a pure lanthanum strontium titanium oxide sample; **b** band contrast image of 0.1 wt % graphene/lanthanum strontium titanium oxide. **c** ZT at room temperature and at 750 °C as a function of the graphene concentration. Reproduced with permission from Ref. [187]. Copyright 2015, American Chemical Society. **d** Lattice thermal conductivity and (inset) total thermal conductivity of the Al-doped ZnO and AZO/rGO samples. Reproduced with permission from Ref. [188]. Copyright 2015, American Chemical Society. **e** Thermoelectric properties of the Bi_2_Te_3_/GQDs samples with different GQD contents. Reproduced with permission from Ref. [189]. Copyright 2017, American Chemical Society

Graphene has been widely investigated in many fields due to its excellent properties. Its high Seebeck coefficient (about 30 mV K^−1^), ultrahigh electrical conductivity (> 14 S cm^−1^), large charge-carrier mobility (> 2 × 10^5^ cm^2^ (Vs) ^−1^), and extraordinary electronic transport properties make it an ideal candidate for the fabrication of high-performance thermoelectric materials. In the past years, graphene was chosen as a composite filler to fabricate high-performance bulk thermoelectric composites. In 2015, Lin et al. fabricated a graphene/lanthanum strontium titanium oxide (LSTO) composite [187]. Upon the addition of a small amount of graphene, the thermal operation window of LSTO decreases to room temperature. As shown in Fig. 9, the highest ZT value is about 0.42 at room temperature and 0.36 at 750 °C was obtained when a quantity of 0.6% of graphene was added into the samples. Chen et al. successfully modulated the carrier concentrations, the electrical conductivity, and the thermal conductivity by adding reduced graphene oxide into the Al-doped ZnO [188]. Since the carrier concentration and the electrical conductivity of graphene oxide are much larger than those of ZnO, the nanocomposite exhibits an enhanced electrical conductivity when compared to pristine Al-doped ZnO. More importantly, although the thermal conductivity of graphene oxide is much higher than that of ZnO and Al-doped ZnO, the total thermal conductivity and the lattice thermal conductivity show an obvious decrease, upon the addition of graphene. These results are shown in Fig. 9d. Li et al. [189] found that by adjusting the density and the dispersion manner of graphene in the bulk matrix, the thermoelectric performance of the composite could be further enhanced. Upon an increase in the graphene amount, the Bi_2_Te_3_ density at the interface increases. Moreover, the high density of the heterojunction interface may enhance carrier scattering and decrease its mobility, thus leading to a decrease in the electrical conductivity and in the thermal conductivity of the compound. In addition, the size of the dopant shows a pronounced effect on the carrier and phonon-transport characteristics. For example, as reported in Li’s paper, the Bi_2_Te_3_/graphene composite exhibits a highest ZT value (~ 0.55) at 425 K as the size of the graphene reaches 20 nm, as shown in Fig. 9e [189]. The same result was also reported for other bulk thermoelectric material systems, such as PbTe/graphene composite [190], Cu_2_SnSe_3_/graphene [191], CuInTe_2_/graphene [192], CoSb_3_/graphene [193], SnSe/MoS_2_/graphene [194], and Bi_0.5_Sb_1.5_Te_3_/graphene [195].

**Fig. 10:**
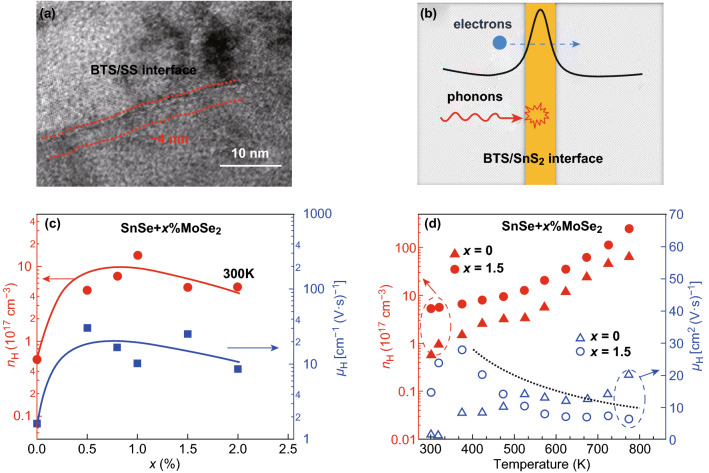
**a** HRTEM image of the Bi_2_Te_2.7_Se_0.3_/SnS_2_ bulk; **b** mechanisms that contribute to the high ZT value of the Bi_2_Te_2.7_Se_0.3_/SnS_2_ nanocomposites. Reproduced with permission from Ref. [196]. Copyright 2017, Elsevier. **c** Carrier concentration and mobility of the SnSe/MoSe_2_ composites at 300 K as a function of the MoSe_2_ content. **d** Temperature dependence of carrier concentration and of the carrier mobility of SnSe/MoSe_2_. Reproduced with permission from Ref. [197]. Copyright 2017, IOP Publishing

Besides graphene, TMDCs have also been widely used as efficient composite fillers. In 2017, Li et al. [196] partially decoupled the electrical conductivity, the Seebeck coefficient, and the thermal conductivity by adding 2D SnS_2_ nanosheets into a Bi_2_Te_2.7_Se_0.3_ matrix. As shown in Fig. 10a, the SnS_2_ nanosheets homogenously assemble onto Bi_2_Te_2.7_Se_0.3_ grain boundaries and formed a nanoscale heterojunction interface. A ZT of 0.93 at 450 K was measured. Such high value was attributed to the optimized carrier and phonon-transport characteristics induced by the SnS_2_/Bi_2_Te_2.7_Se_0.3_ interface. Huang et al. [197] reported a high ZT value for a polycrystalline SnSe sample via the fabrication of a MoSe_2_/SnSe composite. Upon the addition of 2D MoSe_2_ into the SnSe matrix, both the carrier concentration and the carrier mobility are significantly improved when compared to pure polycrystal SnSe, as shown in Fig. 10c, d. For the MoSe_2_/SnSe composite, the carrier concentration is about one order of magnitude higher than that of pure SnSe. Moreover, the carrier mobility of the composite shows an impressive enhancement. This phenomenon may be related to the carrier scattering, due to the energy barrier introduced at the heterojunction interface.

### 2D Materials in Polymer Thermoelectric Materials

As a potential high-performance thermoelectric material, conductive polymers exhibit unique advantages such as a low thermal conductivity, flexibility, lightweight, and efficient solution processability [9, 198–202]. However, more work needs to be done to further improve their thermoelectric performance to use them in flexible and wearable thermoelectric devices. The power factor for typical conductive polymer thermoelectric materials is in the 10^−4^ to 10^2^ μW mK^−2^ range at room temperature. This value is about 3 orders of magnitude lower than those of traditional inorganic thermoelectric materials [199, 203, 204]. According to the theoretical and experimental achievement, the fabrication of composite blends is an efficient way to improve the thermoelectric performance of conductive polymers.

In the last decade, a number of investigations have been reported to improve the thermoelectric performance of conductive polymers by adding a nanostructure filler. Polymer blends fabricated by employing highly electrical conductive constituents and high-Seebeck-coefficient constituents show an improvement in both their Seebeck coefficient and electrical conductivity. As previously discussed, 2D materials possess excellent electrical and mechanical properties, which are suitable to fabricate high-performance composite thermoelectric materials. In a simple 2D materials/polymer composite system, its thermoelectric performance of the composite can be evaluated based on either a serially or parallel connected model, if the interfaces are neglected [205, 206]. The simulation results indicate that the Seebeck coefficient and the electrical conductivity cannot exceed those of their individual components [207, 208]. However, recent advances in the theory have proven that the introduction of an energy filtering effect at the interface can overcome this limitation and can be experimentally proven [206, 207, 209].

**Fig. 11:**
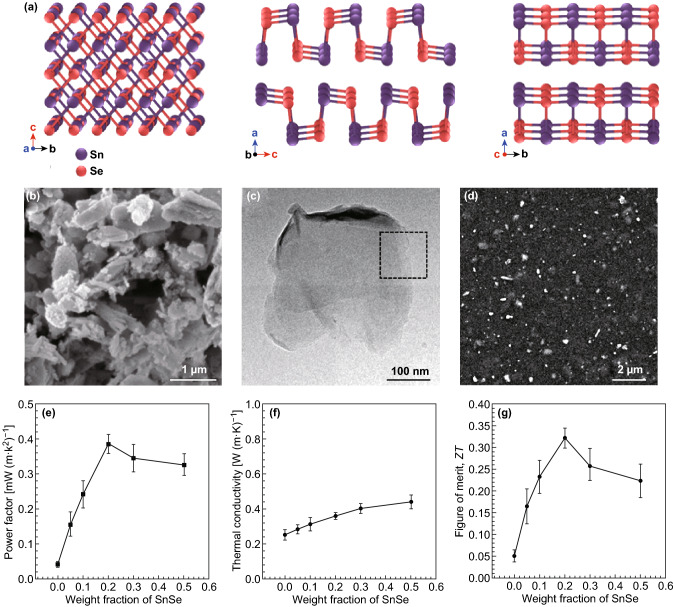
**a** Crystal structures of SnSe along the *a*-*, b*-, and *c*- axial directions. **b** SEM morphology and **c** TEM image of SnSe nanosheets. **d** SEM of the SnSe/PEDOT:PSS composites with SnSe a content of 50%. **e** Power factor, **f** thermal conductivity, and **g** ZT value of the SnSe/PEDOT:PSS composites. Reproduced with permission from Ref. [205]. Copyright 2016, American Chemical Society

**Fig. 12:**
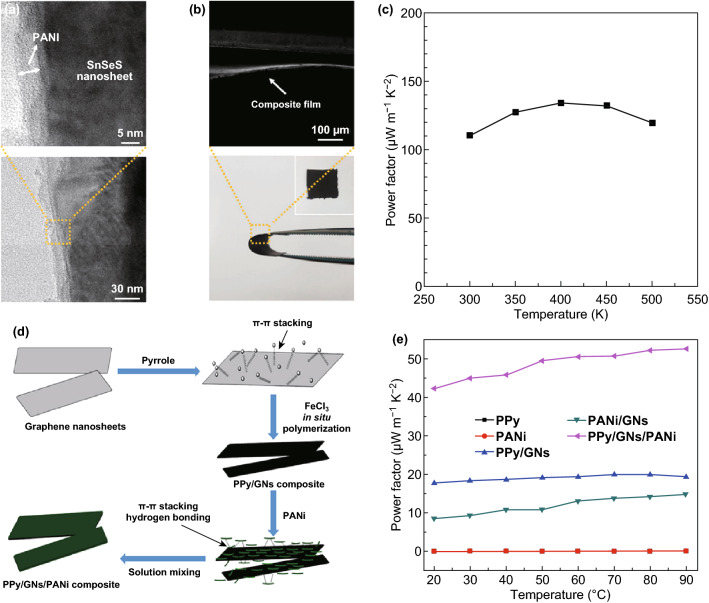
**a** TEM image of the SnSe_0.8_S_0.2_ nanosheets, **b** SEM image of the PANI-coated SnSe_0.8_S_0.2_ film and **c** power factor of the PANI-SnSeS nanosheet/PVDF composite film with a PANI-SnSeS nanosheet-to-PVDF ratio of 2:1 as a function of temperature. Reproduced with permission from Ref. [212]. Copyright 2018, American Chemical Society. **d** Schematic process of the polypyrrole/graphene/polyaniline ternary nanocomposite and **e** its power factor. Reproduced with permission from Ref. [220]. Copyright 2017, American Chemical Society

2D materials with a high Seebeck coefficient have been widely employed as efficient fillers to enhance the thermoelectric performance of polymer blends. 2D materials were added into the polymer blends to increase their Seebeck coefficient. For instance, the group IV metal chalcogenides (e.g., SnSe, SnS) can be exfoliated into 2D materials. The crystal structures of SnSe along different directions at 300 K are shown in Fig. 11a. The strong Sn–Se bonds along the *b*–*c* planes are connected with the weak Sn–Se bonds along the *a*-axis of SnSe, indicating that bulk SnSe can be easily exfoliated into 2D ultrathin materials along the a-direction. Ju et al. [205] prepared SnSe nanosheets via hydrothermal lithium-intercalation, which was then followed by an exfoliation process from the SnSe powders. During the intercalation process, ethylene glycol acts as both the solvent and the reducing agent. The morphology of the exfoliated SnSe nanosheets is shown in Fig. 11b–d. Their thickness is about 3.4 nm. The Seebeck coefficient of pure SnSe at room temperature is higher than 520 μV K^−1^, whereas for PEDOT:PSS is about 30 μV K^−1^. As the SnSe nanosheets are dispersed into the PEDOT:PSS solution, the measured Seebeck coefficient increases with the increase in the weight fraction of SnSe. The increase in the power factor to 386 μW mK^−2^ is induced by the substantial increases in the Seebeck coefficient when compared to the reduction in the electrical conductivity of the material. Other 2D group IV metal chalcogenides have also been fabricated and added as fillers to enhance the thermoelectric performance of the composites [210–213]. Ju et al. [212] coated the SnSeS nanosheet by polyaniline (PANI) and then used it as a filler to fabricate the composite. The PANI-coated SnSeS nanosheet added into polyvinylidene fluoride (PVDF) exhibits a maximum power factor of 134 μW mK^−2^ at 400 K, as shown in Fig. 12a. SnS has also been chosen as a filler to improve the properties of polymer thermoelectric materials. Recently, Cheng et al. [214] have fabricated a SnS/PEDOT:PSS composite and showed that its Seebeck coefficient increases upon the increase in its SnS content. In 2019, Ju et al. fabricated PANI-coated porous SnS nanosheets and characterized its thermoelectric properties [214]. An outstanding ZT value of 0.078 at 450 K was obtained by adjusting the PANI coating layer.

Besides the group IV metal chalcogenides, TMDCs have also been widely studied as high-performance thermoelectric materials. TMDCs have gained considerable attention as a potential thermoelectric material in recent years due to their low thermal conductivity (0.1–1 W mK^−1^) and their large in-plane mobility (200–500 cm (Vs) ^−1^). Recently, several theoretical and experimental studies on the thermoelectric performance of TMDCs have been reported. Although the thermoelectric performance of the TMDCs-based bulk materials is limited due to their poor electrical conductivity, TMDCs with their large Seebeck coefficient are an ideal composite filler to fabricate high-performance polymer blends. In 2010, Zhang et al. [215] incorporated both a *n*-type and a *p*-type Bi_2_Te_3_ into a PEDOT:PSS solution and successfully fabricated both *p*-type and *n*-type polymer composite materials. However, by adjusting the morphology of the nanostructure, the thermoelectric performances of these composites were further enhanced. Du et al. [216] fabricated a Bi_2_Te_3_ nanosheet/PEDOT:PSS thin-film composite via a simple coating process. With the addition of Bi_2_Te_3_ nanosheets, the Seebeck coefficient and the electrical conductivity of the composite increase simultaneously. Generally, in this kind of composite materials, a 2D material filler is chosen to improve the Seebeck coefficient, thus leading to a high power factor. In 2016, Jiang et al. [217] fabricated a high-performance MoS_2_/PEDOT:PSS thin film via a vacuum filtration process. By the addition of a small amount of liquid-phase exfoliated MoS_2_ nanosheets into a PEDOT:PSS solution, the thermoelectric properties of the PEDOT:PSS-based thin film were enhanced significantly. Several 2D nanostructures including nanosheet, nanoparticles, nanowire, and nanobarbell were reported to enhance the thermoelectric performance of the films [205, 211, 212, 215–219].

**Fig. 13:**
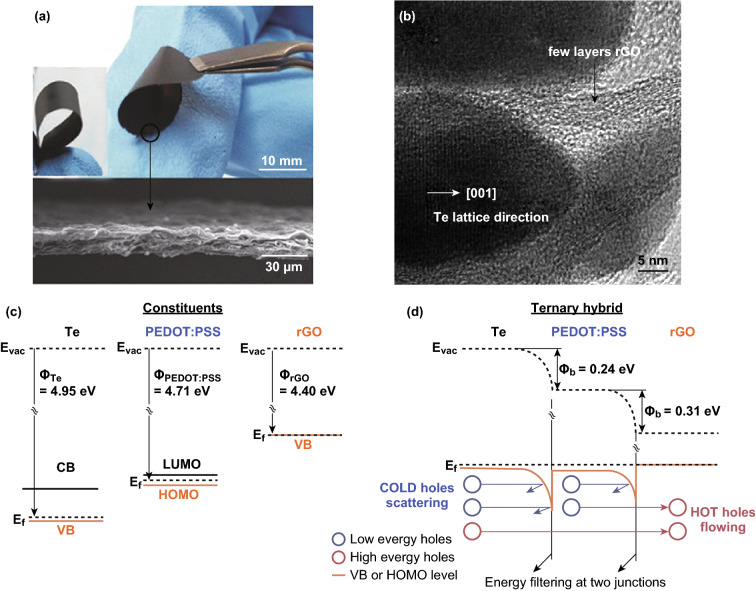
**a** Photographs and cross-sectional SEM images of the flexible rGO/PEDOT:PSS/Te hybrid paper. **b** HRTEM images of the rGO/PEDOT:PSS/Te hybrid composite. **c** Energy diagram of the rGO/PEDOT:PSS/Te heterojunctions. **d** Energy filtering effects at two junctions. Reproduced with permission from Ref. [208]. Copyright 2016, Wiley–VCH

Moreover, the addition of a high-Seebeck-coefficient component, high-electrical-conductivity component was also employed to enhance the electrical properties of the hybrid composite, leading to an enhanced power factor. However, 2D materials with a high electrical conductivity, such as graphene and reduced graphene oxide, have also been widely studied. Due to their relatively low electrical resistivity, this group of 2D materials is usually employed to enhance the electrical conductivity of the composite. As shown in Fig. 12c, Wang et al. [220] fabricated a polypyrrole/graphene/polyaniline ternary nanocomposite via a simple in situ polymerization process. Due to the ultrahigh electrical conductivity of graphene, this ternary composite exhibits at least two magnitude higher Power factors than that of PANI and PPy. As shown in Fig. 13, Choi et al. [208] fabricated a novel Graphene/PEDOT:PSS/Te composite. The electrical conductivity of such composite is about 15 times higher than that of PEDOT:PSS/Te although the Seebeck coefficient is almost identical. The significant increase in the electrical conductivity of the ternary composite is ascribed to the carrier scattering at the double interface, as shown in Fig. 13c, d. This work shows that graphene may be an ideal composite filler to optimize the thermoelectric performance of polymers. Inspired by these pioneer works, several investigations, including graphene/P_3_HT, rGO/PEDOT:PSS, PANI/graphene and graphene/CNT, have reported the use of graphene or reduced graphene oxide into a conductive polymer [43, 59, 221–226].

Besides their use in the enhancement of either the Seebeck coefficient or the electrical conductivity, 2D material-based composites can be employed as electrodes to fabricated high-performance flexible devices. Jiang et al. [227] prepared a graphene/polyethyleneglycol composite, which was used for heat collection and transport. The presence of highly conductive graphene in this composite introduces several conductive pathways for heat transfer and acts as a highly thermal conductive reservoir of phase-change materials for thermal energy collection, storage, and release. Moreover, this work opens a new door to fabricate novel thermoelectric materials and to realize high-performance flexible thermoelectric devices.

## Thermoelectric Properties of Single- or Few-layer 2D Materials

In the past years, a significant effort has been made to improve the thermoelectric efficiency of the 2D materials. The most efficient ways to enhance their thermoelectric performance is developing novel materials and novel nanostructures. As previously mentioned, 2D layered materials have been widely investigated either by fabricating bulk/thin film or by manufacturing hybrid composites as fillers. Moreover, studying the thermoelectric properties of single- or few-layer materials via the fabrication of micro–nanodevices is another important field in the thermoelectric research environment. The thermoelectric properties of the bulk and of individual single-layer materials may differ and this may provide a deeper understanding of the physical mechanism behind their thermoelectric effect. For example, Kumar et al. [149] simulated the thermoelectric properties of bulk and monolayer MoSe_2_ and WSe_2_ by using the first-principles calculations and the semiclassical Boltzmann transport theory. The calculation results proved that the electrical conductivity, the Seebeck coefficient, and the thermal conductivity of the monolayer materials are extremely different from the bulk ones. These results allow one to optimize the thermoelectric properties based on the unit materials.

Decoupling the Seebeck coefficient, the electrical conductivity, and the thermal conductivity is a challenging problem to solve. However, the different mean free paths of electrons and phonons provide the possibility to decouple the electrical properties and the thermal properties, which are governed by the Wiedemann–Franz law [228]. In detail, several kinds of low-dimensional nanostructure material systems may enable high-speed transmittance of electrons by impeding the propagation of acoustic phonons. As a result, these compounds exhibit a suppressed thermal conductivity, whereas their electrical conductivity remains almost identical [93, 228, 229]. When the size of the material is small enough to influence its band structure, the profile of the density of states (DOS) can evolve into sharp shapes at the band edges due to the quantum confinement effect of these nanomaterials [228]. This phenomenon may increase the Seebeck coefficient, since this is strongly related to the change rate of the DOS near the Fermi energy [93, 230].

**Fig. 14:**
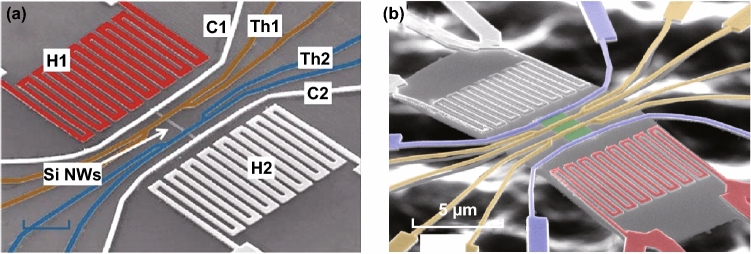
SEM images of **a** non-suspended thermoelectric micro-device platform and **b** suspended thermoelectric micro-device platform. Reproduced with permission from Ref. [235]. Copyright 2008, Springer Nature

The typical structure of the micro–nanodevices, which are used to measure the thermoelectric performance of a single- or multilayer 2D material, is shown in Fig. 14. Two main micro-devices are currently used: a typical micro-device for the Seebeck coefficient and the electrical conductivity measurements and a suspended micro-device to determine the ZT value. The thermoelectric properties of nanostructure materials with different morphologies, including nanowires, nanosheets, nanoplates, and single- or multilayer 2D materials, can be measured via these micro-devices.

In a typical platform, the micro-device is usually patterned onto a silicon substrate which is coated with an insulating SiO_2_ layer. As shown in Fig. 14, one or two heaters are employed to build a temperature gradient through the nanosample. The samples are usually placed onto the substrate via a simple drop-casting process from a solution, which contains a suspension. Then, the metal electrodes are deposited onto the sample via an electron beam lithography process. The Au/Cr metal contact with the sample is fabricated and serves simultaneously as both an electrode and a thermometer [231]. However, for such micro-devices, since the electrodes and the sample are directly in contact with the substrate, only the Seebeck coefficient and the electrical conductivity can be measured. In order to evaluate the ZT value of the nanostructure 2D materials, a modification of this micro-device has been developed. As shown in Fig. 14b, the electrode and the micro-heaters are supported by five slender SiN_*x*_ beams which support the platform in vacuum onto the substrate. This micro-device platform allows one to measure the thermal conductivity of the nanomaterials and to calculate their ZT value [49, 232–235]. Under high vacuum, the heat loss of the sample via convection and radiation is negligible. Moreover, the monolithic silicon device minimizes the thermal contact resistances. When a micro-heater is applied to the system, the suspended micro-device in vacuum forces a heat flow across the samples. By carefully measuring the temperature at both sides of the sample and calculating the total amount of heat delivered to the micro-heater, the thermal conductance of the nanostructure 2D materials can be calculated. When combined with the dimensional data of the 2D materials, the thermal conductivity of each 2D material can be obtained based on such well-established thermometric technique [227, 231, 236].

A number of researchers have investigated the thermoelectric properties of individual 2D nanostructure materials for several years. In 2008, two remarkably high ZT values of 0.6 and 1.2 for two samples of highly doped silicon single-crystalline nanowires at near room temperature have been reported independently by Heath and Yang’s group [235, 237]. According to their studies, a suppression of the thermal conductivity was observed due to the rough surface and the reduction of diameter of the sample. By using these microstructure measurement platforms, the thermoelectric properties of many individual nanowires, nanosheets, quantum dot superlattices, and thin films were investigated [227, 231, 233, 234, 236, 238–245]. Recently, the thermoelectric properties of single-layer and multilayer 2D materials have been studied via these microstructure measurement platforms.

**Fig. 15:**
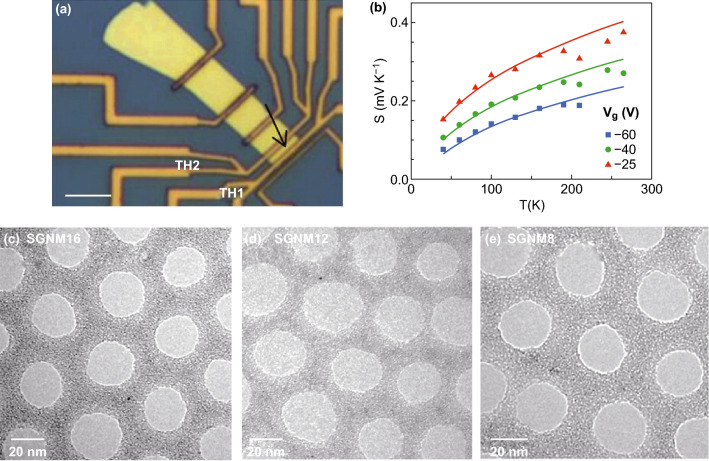
**a** Optical microscope images of the measurement devices prepared onto a 300-nm-thick SiO_2_/Si substrates. **b** Seebeck coefficient of few-layer black phosphorus depending on temperature and gate voltage. Reproduced with permission from Ref. [131]. Copyright 2016, American Chemical Society. **c** TEM image of single-layer graphene nanomeshes with neck width of 16 nm. TEM image of bilayer graphene nanomeshes with neck width of **d** 14 nm and **e** 8 nm. Reproduced with permission from Ref. [249]. Copyright 2017, Elsevier

As previously mentioned, graphene and BP are considered potential high-performance thermoelectric materials and have been widely employed as composite fillers to enhance the thermoelectric properties of the composites. However, the thermoelectric properties of individual sample of graphene and BP have also been studied via these microstructure measurement platforms. Choi et al. [131] measured the thermoelectric performance at various temperatures and at gate electrical fields of BP and then studied its transport characteristics in samples with a thickness of 10–30 nm. The structure of this device is shown in Fig. 15a. The test results prove that the 2D Mott’s variable rang hopping is a dominant mechanism in the thermal and electrical transport in thin BP samples. Saito et al. [134] investigated the gate-tuned thermoelectric performance of BP at low temperature (210 K). By using the electric-double-layer transistor configuration, the Seebeck coefficient of ion-gated BP reached + 510 μV K^−1^ at 210 K. This value is about 1.5 times higher than the value (+ 340 μV K^−1^ at 300 K) of a bulk single crystal.

Several computational simulation results have shown that an ultrahigh thermoelectric performance can be achieved in various graphene nanostructures, including zigzag and armchair graphene nanoribbons, graphene nanomeshes, and graphene superlattices [48–50, 52–55, 64, 246, 247]. These simulation results show that the ZT value can be enhanced by controlling the nanostructure or by employing a proper post-treatment. By controlling the microstructure and the layers of graphene, its thermoelectric efficiency can be greatly improved. Furthermore, by tuning the band structures, a high thermopower and a lower thermal conductivity can be achieved simultaneously due to the dominant effect of the phonon-edge scattering. In addition, these theoretic predictions have been proved via experimental measurements. Xiao et al. [248] found that the Seebeck coefficient of a few-layer graphene sample can be greatly enhanced (> 700 μV K^−1^) after oxygen plasma treatment. Since a graphene monolayer is easy to damage during the plasma treatment process, the oxygen plasma treatment is not suitable. Oh et al. [249] fabricated single- and bilayer graphene nanomeshes with various neck widths via block copolymer self-assembly on graphene, as shown in Fig. 15c–e. Since the patterned graphene nanostructure can induce a dominant phonon-edge scattering and quantum confinements to control the electron and phonon-transport behavior, the bilayer graphene nanomeshes with 8 nm neck widths exhibit an ultralow thermal conductivity and an enhanced ZT value. Anno et al. achieved a ZT value of graphene, which is about 3 times higher than in the case of pristine graphene via defect engineering [66].

Besides graphene and BP, the thermoelectric properties of other 2D materials including 2D tellurium, TMDCs, and group IV–VI compounds were measured via these microstructure measurement platforms. Moreover, the nanopattern effect and the pattern geometry effect on the thermal and the thermoelectric phenomena of nanopatterned 2D materials were characterized [249]. For example, Qiu et al. [228] investigated the thermoelectric properties of 2D tellurium for the first time and reported a ZT value of 0.63 at room temperature. The Seebeck coefficient and the electrical conductivity of 2D Tellurium were measured by using the microstructure measurement platforms as shown in Fig. 14a. The thermal conductivity was measured via the micro-Raman method. Pettes et al. [234] investigated the thermoelectric properties of a Bi_2_Te_3_ nanoplate with the thickness in the 9–25 nm range by using a suspended micro-device. When the thickness increases, the Bi_2_Te_3_ nanoplate exhibits a suppressed Seebeck coefficient, whereas both the electrical conductivity and the thermal conductivity are decreased. However, this result is mostly related to the surface band bending and the diffuse surface scattering of the electrons and the phonons in the nanoplates. The thermoelectric signature of 2D materials, such as the WSe_2_ single crystal, the single-layer SnSe, the SnTe nanoplate, and the Cd_3_As_2_ superlattice, was also studied by using the microstructure measurement platform [243, 244, 250, 251].

**Fig. 16:**
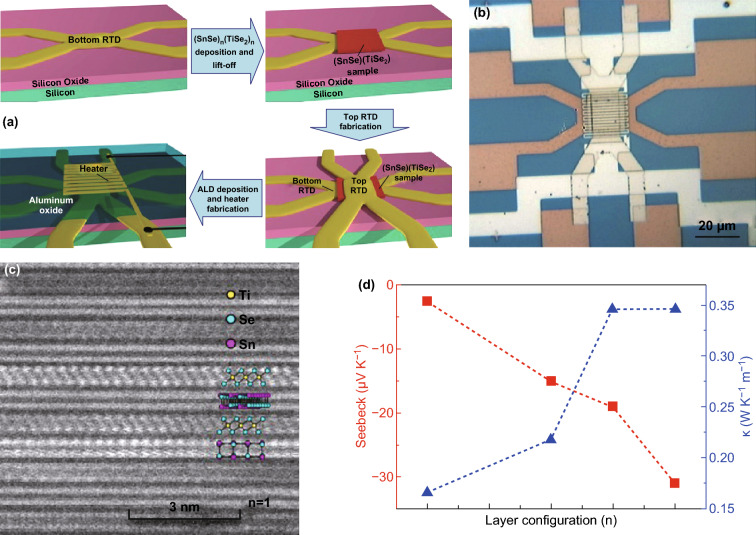
**a** Schematic diagram of the device fabrication process, and **b** its optical microscope image. **c** STEM images of the (SnSe)_*n*_(TiSe_2_)_*n*_ thin film (*n* = 1). **d** Cross-plane Seebeck coefficient and thermal conductivity of the (SnSe)_*n*_(TiSe_2_)_*n*_ thin film with different layer configurations. Reproduced with permission from Ref. [252]. Copyright 2017, American Chemical Society

As previously shown, the thermoelectric properties of individual 2D materials were investigated along the plane direction. As is well known, 2D materials usually exhibit a high anisotropy along the perpendicular direction and in-plane direction. However, it is difficult to measure the thermal conductivity, the Seebeck coefficient, and the electrical conductivity of single-layer and multilayer 2D materials along the perpendicular direction. Recently, several researchers have fabricated cross-plane thermoelectric devices to investigate the cross-plane thermoelectric properties of 2D materials. Figure 16a, b shows an optical image of a completed device and the schematic diagram of its fabrication process. To manufacture such cross-plane device, a bottom/top metal 4-probe resistance temperature detector and a serpentine metal heater are patterned onto the silicon oxide/silicon substrate via electron beam lithography. This process is then followed by metal deposition of a metal electrode. Before the top resistance temperature detector is deposited, the sample should be capped with an insulating film of Al_2_O_3_. By using such microstructure measure platform, the Cronin’s group studied the thermoelectric properties of SnSe_2_ and (SnS(e)_*n*_(TiS(e)_*n*_ (*n* = 1, 3, 4, 5) thin-film layer materials along cross-plane direction [252, 253]. The Seebeck coefficient and the thermal conductivity of the layered structure materials are strongly dependent on the number of layers. Moreover, several researchers found that the Seebeck coefficient, the electrical conductivity, and the thermal conductivity along cross-plane direction are very different from the properties along the in-plane direction. This is in agreement with the properties of bulk thermoelectric materials based on 2D materials (such as SnSe, Bi_2_Te_3_, and Sb_2_Te_3_). For example, Li et al. found that when the number of layers decreases from 5 to 1, the cross-plane Seebeck coefficient decreases from − 31 to − 2.5 μV K^−1^, whereas the cross-plane thermal conductivity decreases from 0.35 to 0.15 W mK^−1^ (Fig. 16c, d), due to an increased interfacial phonon scattering [252]. Juang et al. [254] proposed a cross-plane micro-device onto a silicon substrate to measure the thermoelectric properties of the vertical graphene/gold nanoparticles heterostructure along the perpendicular direction. Since the Au nanoparticles can further inhibit the phonon transport and enhance the electrical conductivity along the perpendicular direction, a high ZT value larger than 1.0 (at room temperature) can be achieved for a single-layer graphene. Chen et al. [255] fabricated a graphene/hexagonal boron nitride/graphene heterostructure device and measured its thermoelectric transport properties. The top and bottom graphene surfaces in this device work as both an electrode and a micro-heater to induce a temperature gradient. The measured thermoelectric properties are useful to understand the thermoelectric component in the cross-plane behavior of emerging 2D heterostructure devices.

The development of micro–nanoprocessing techniques enables us to fabricate micro-devices to investigate the thermoelectric properties of individual 2D nanostructure materials. The study of the thermoelectric transport characteristics of individual 2D materials is pivotal to understand its physical mechanism. The massive proliferation of 2D materials, such as TMDCs, BP, MXenes, graphene, and Xene, offers new opportunities to engineer such compounds and to fabricate high-performance thermoelectric devices. The device structures reviewed in this section serve as a general approach to characterize the in-plane and cross-plane transport properties of 2D materials.

## Thermoelectric Materials Combined with Photodetection

Thermoelectric materials have been widely used as power generators, cooling devices, and sensors. Recently, they have been found to work as power supplies and can be used to develop highly efficient and wearable self-powered electronics devices. As a group of developing materials, 2D materials have been widely employed as optoelectronic devices, including in the manufacturing of photodetectors and photovoltaic devices. When irradiated by light, these devices convert photons into electric current due to separation of the excited electron–hole pair via a built-in electric field. The development of low-cost and high-performance broadband photodetectors is the key to fulfill different application requirements. The photothermoelectric effect, which is based on the Seebeck effect, enables the device to generate a photocurrent due to the temperature gradient induced by the absorbed light on an electric voltage. Moreover, this temperature gradient would be generated across the materials due to light irradiation and the photocurrent is generated by the photothermoelectric effect. Self-power photodetectors are promising devices, which can be employed in a large variety of application including sensing, environmental monitoring, night vision, and astronomy.

2D materials, such as graphene and BP, have been widely used as photothermal agents due to their efficient photothermal conversion efficient [122, 256–259]. Gabor found that upon heating a junction consisting of a single graphene sheet by shining laser onto it, a thermoelectric voltage is generated across the junction [260]. However, this kind of photothermoelectric effect was found in other 2D materials system, as well [51, 71, 126, 261–264]. As predicted by Basko, this effect can be potentially exploited in novel optoelectronic devices [70].

In 2011, Kraemer et al. [265] reported a novel solar thermal flat panel, which converts thermal energy into electric power based on the Seebeck effect and on the high thermal concentration. The peak efficiency of such solar thermoelectric generator is close to that of a common solar cell (4.6%). However, the total power conversion efficiency of this device is limited by the photothermal and thermoelectric efficiency [56, 265].

Besides their use as solar energy converters, 2D thermoelectric materials also can be employed in the fabrication of high-performance self-power photodetectors. When compared to other photodetection mechanisms, such as photoconduction, photovoltage, and bolometry, the photothermoelectric effect achieves a broadband detection without an external bias at room temperature. A photodetector based on the photothermoelectric effect mechanism exhibits a responsivity, which is related to the light-induced temperature gradient and to its Seebeck coefficient. The temperature gradient is determined by the absorption, heat capacity, and the photothermal conversion efficiency of the material.

**Fig. 17:**
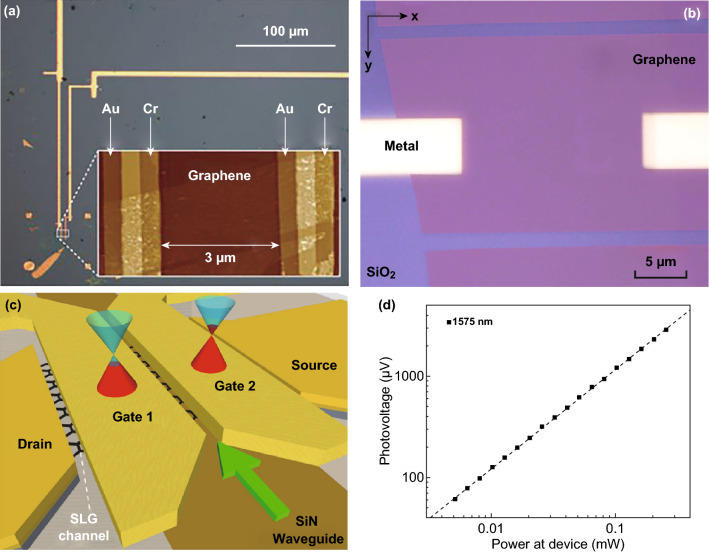
**a** Optical micrograph and atomic force microscopy of graphene photodetector with asymmetric metal electrodes. Reproduced with permission from Ref. [266]. Copyright 2014, Springer Nature. **b** Optical micrograph of the graphene-based photodetector devices. Reproduced with permission from Ref. [61]. Copyright 2014, American Chemical Society. **c** Scheme of the single-layer graphene photodetector SiN waveguide. The green arrow indicates the light propagation direction. **d** The photovoltage *V*_ph_ dependence of optical power. Reproduced with permission from Ref. [267]. Copyright 2019, American Chemical Society

Graphene and BP are the most widely studied 2D materials and have attracted a large attention. In 2010, Xu et al. [51] found that the photothermoelectric effect has a pronounced effect into the photocurrent generation process at the graphene interface in field-effect transistors. This phenomenon has several positive benefits in the design of high-performance optoelectronics devices based on graphene. As shown in Fig. 17a, Cai et al. [266] fabricated a simple thermoelectric terahertz photodetector based on graphene, which exhibits an excellent sensitivity exceeding 10 V W^−1^ (700 V W^−1^) at room temperature and noise-equivalent power less than 1100 pW Hz^−1/2^ (20 pW Hz^−1/2^), referenced to the incident (absorbed) power. The performance of such graphene-based thermoelectric terahertz photodetectors can compete with the best room-temperature terahertz detectors to develop optimally coupled devices. Echtermeyer et al. [61] investigated the influence of the wavelength on the performance of metal-graphene-metal photodetectors via polarization-dependent measurements. The device that used in this study is shown in Fig. 17b. As shown in Fig. 17c, Muench et al. report a compact, photothermoelectric-based, waveguide- integrated, plasmonic enhanced graphene photodetector for telecom wavelengths operating at zero dark current. Owing to the voltage generated by the photothermoelectric, the graphene photodetector exhibits an external responsivity ~ 12.2 V W^−1^ and a 3 dB bandwidth ~ 42 GHz [267]. The photovoltage (*V*_ph_) generated by the thermoelectric current as for the Seebeck effect dependence on power is shown in Fig. 17d. The linear response indicates a power-independent external voltage responsivities in the tested optical power range [267].

As graphene, BP has also been studied as a high-performance photothermoelectric photodetector. Hong et al. [126] investigated the electrical transport and optoelectronic properties of FETs manufactured from a few-layer BP sample. Their results reveal that the photocurrent signals at the BP–electrode junctions can be mainly attributed to the photovoltaic effect in its the off-state, whereas the photothermoelectric effect occurs during its on-state. Leong et al. [268] fabricated a device based on exfoliated BP nanoflakes with a responsivity of 1 mV W^−1^ for a 2.5-THz beam with a diameter of 200 μm. In this case, the photothermoelectric effect was found to be the primary source of the THz photosignal.

**Fig. 18:**
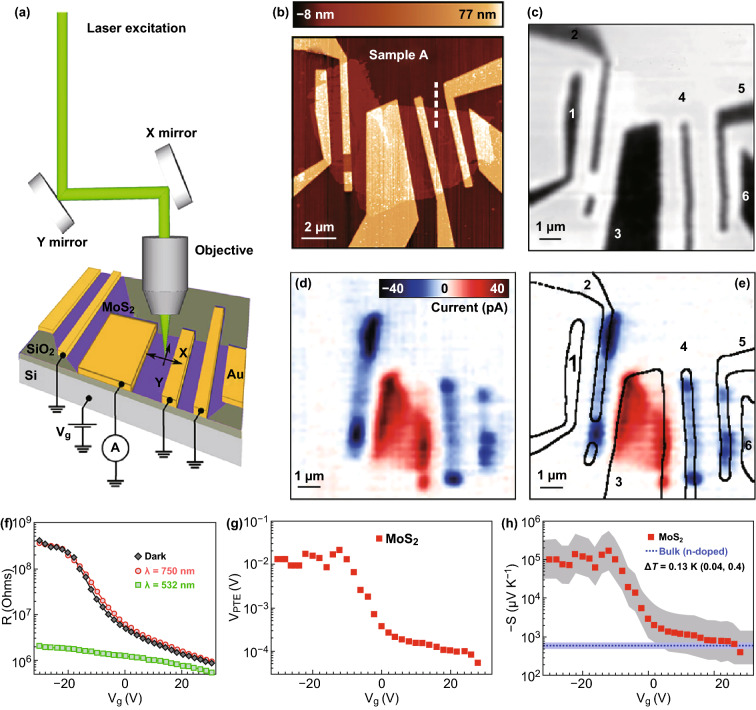
**a** Schematic of the scanning photocurrent microscopy setup. **b** AFM image of the micro-devices with a MoS_2_ flake. **c** Spatial map of the intensity of the reflected light from the device. **d** Photocurrent image of the MoS_2_ FET. **e** Superposition of the photocurrent map and the contours of the electrodes, which were obtained from the light reflection map. **f** Resistance and **g** photothermoelectric voltage of the MoS_2_ device as a function of the gate voltage in its dark state and with the laser spot placed on the MoS_2_/electrode interface. Estimated Seebeck coefficient as a function of the gate voltage. Reproduced with permission from Ref. [71]. Copyright 2013, American Chemical Society

The photothermoelectric effect of several 2D TMDC materials, such as MoS_2_, WSe_2_, and Bi_2_Se_3_, has also been widely investigated [71, 261, 262, 264]. Buscema et al. [71] studied the photoresponse of MoS_2_ monolayer FETs via scanning photocurrent microscopy. The device and results are shown in Fig. 18. To summarize, the photocurrent generation in a MoS_2_ monolayer is dominated by the photothermoelectric effect. The separation of the photoexcited electron–hole pairs across the Schottky barriers at the MoS_2_/electrode interfaces only plays a marginal role. As shown in Fig. 18h, a large and controllable Seebeck coefficient in the − 1 × 10^5^ to −4 × 10^2^ μV K^−1^ range was observed in the case of MoS_2_ monolayer. However, the mechanism behind the generation of the photocurrent is different when compared to other 2D TMDC materials. In order to deeply understand such physical mechanism, Groenendijk et al. [262] studied the photocurrent in a WSe_2_ sample by fabricating a double-gated WSe_2_ device and by applying varied gate voltages and illumination power. The results show that the photocurrent of the WSe_2_-based devices (for both the PN and NP configurations) is mainly generated by the photovoltaic effect. Moreover, a maximum responsivity of 0.7 mA W^−1^ at 532-nm illumination was obtained. In the PP configurations devices, the photocurrent mainly is generated by the photothermoelectric effect and the intensity is about 2 times larger than that generated by the photovoltaic effect. Due to the strong optical absorption caused by an asymmetry in flake thickness or by the optical absorption of the electrodes, a sizable temperature gradient can be generated across the 2D material-based devices. In addition, the photothermoelectric effect plays a significant role in such devices and can be used to develop high-performance self-power optoelectronic devices.

Photothermoelectric photodetectors have exhibited their advantages in developing low-cost, uncooled ultrafast and ultrabroadband photodetectors, and their performance has been significantly enhanced owing to the development of low- dimensional materials and the micro–nanofabrication technology. However, due to the limited effective active surface area, the saturation effect and relatively low practical Seebeck coefficient at room temperature limit the temperature gradient in the case of exfoliated or single-crystal 2D materials. For this reason, no high voltage can be generated. Although tremendous progress has been made in recent years, more work needs to be done to further optimize the performance of 2D material-based photothermoelectric photodetectors.

## Conclusions and Outlook

During the last decades, a significant progress has been achieved in the development of thermoelectric materials. As new members of the thermoelectric material family, 2D materials have attracted a large attention and many milestone investigations have been reported. Due to their unique electronic, thermal, and mechanic properties, 2D thermoelectric materials are expected to be the next-generation high-performance thermoelectric materials.

In this review, the thermoelectric properties of various 2D materials, such as TMDCs, graphene, BP, group IVA–VA compounds, MXene, and their nanocomposites, were illustrated in detail. Moreover, the state-of-the-art of theoretical and experimental data were presented to elucidate the relation among various factors which determine the thermoelectric properties of these materials. Although a tremendous progress has been achieved in the past few years, these properties still need to be improved for their practical application as thermoelectric devices. Here, some major outlooks are presented to address this issue:

1. The thermoelectric performance of 2D materials is still much lower then for conventional bulk thermoelectric materials. A precise chemical doping during the fabrication and the functionalization of these composites must be performed to achieve a high value of ZT value. This remains a challenging issue.
2. The electronic and phonon-transport mechanisms of 2D materials are still obscure. Although the power factors of individual single- and few-layer 2D materials have been investigated and a series of possible mechanisms have been proposed, it is still impossible to decouple these factors. The current knowledge of the effects of the interfacial electronic and the phonon-transport mechanism on the thermoelectric performance is still limited. Their deeper understanding should guide the design of novel thermoelectric composite and further improve their thermoelectric properties.
3. The thermal conductivity of 2D materials is too high, and this limits their thermoelectric performance. The inherent thermal conductivity of the most common 2D materials, including graphene, TMDCs, and BP, is much higher than for the traditional semiconductor thermoelectric materials. Their structural modifications, as well as the introduction of structural defects and the control of their elemental components, are likely to have a positive effect on the thermal conductivity of these materials.
4. The fabrication realization and the characterization of 2D material-based thin films are still challenging. With the increase in the demand of wearable and portable flexible devices, thin-film thermoelectric materials have received a wide attention. Although they have been studied for very short time when compared to bulk thermoelectric materials, they are considered the next generation of thermoelectric materials for practical applications. Due to their excellent electronic and mechanical properties, several researchers have tried to fabricate thin-film thermoelectric materials by using 2D nanomaterials and their composites. However, the measurements of the thermal conductivity of thin films are still challenging.
5. It is of pivotal importance to achieve a facile production of high-quality and scalable 2D material. This is currently an active research topic. Until now, many methods have been employed to fabricate 2D materials, such as chemical vapor deposition, mechanical exfoliation, atomic layer deposition, molecular beam epitaxy, physical vapor deposition liquid exfoliation, and mechanical exfoliation. However, it is still challenging to manufacture such materials with a controlled structure via scale-up methods.
